# CRISPR-Cas9 immune-evasive hESCs are rejected following transplantation into immunocompetent mice

**DOI:** 10.3389/fgeed.2024.1403395

**Published:** 2024-05-28

**Authors:** Henriette Reventlow Frederiksen, Alexandra Glantz, Kåre Kryger Vøls, Søren Skov, Pernille Tveden-Nyborg, Kristine Freude, Ulrik Doehn

**Affiliations:** ^1^ Department of Veterinary and Animal Sciences, Faculty of Health and Medical Sciences, University of Copenhagen, Copenhagen, Denmark; ^2^ Cell Therapy Research, Novo Nordisk A/S, Maaloev, Denmark

**Keywords:** universal cell line, xenogeneic transplantation, CRISPR-Cas9 editing, transgene insertion, immune rejection, human embryonic stem cells

## Abstract

Although current stem cell therapies exhibit promising potential, the extended process of employing autologous cells and the necessity for donor–host matching to avert the rejection of transplanted cells significantly limit the widespread applicability of these treatments. It would be highly advantageous to generate a pluripotent universal donor stem cell line that is immune-evasive and, therefore, not restricted by the individual’s immune system, enabling unlimited application within cell replacement therapies. Before such immune-evasive stem cells can be moved forward to clinical trials, *in vivo* testing via transplantation experiments in immune-competent animals would be a favorable approach preceding preclinical testing. By using human stem cells in immune competent animals, results will be more translatable to a clinical setting, as no parts of the immune system have been altered, although in a xenogeneic setting. In this way, immune evasiveness, cell survival, and unwanted proliferative effects can be assessed before clinical trials in humans. The current study presents the generation and characterization of three human embryonic stem cell lines (hESCs) for xenogeneic transplantation in immune-competent mice. The major histocompatibility complexes I- and II-encoding genes, B2M and CIITA, have been deleted from the hESCs using CRISPR-Cas9-targeted gene replacement strategies and knockout. B2M was knocked out by the insertion of murine CD47. Human-secreted embryonic alkaline phosphatase (hSEAP) was inserted in a safe harbor site to track cells *in vivo.* The edited hESCs maintained their pluripotency, karyotypic normality, and stable expression of murine CD47 and hSEAP *in vitro*. *In vivo* transplantation of hESCs into immune-competent BALB/c mice was successfully monitored by measuring hSEAP in blood samples. Nevertheless, transplantation of immune-evasive hESCs resulted in complete rejection within 11 days, with clear immune infiltration of T-cells on day 8. Our results reveal that knockout of B2M and CIITA together with species-specific expression of CD47 are insufficient to prevent rejection in an immune-competent and xenogeneic context.

## 1 Introduction

The transplantation of cells for therapeutic purposes and organ transplants is presently constrained by the risk of host-induced rejection of donor material, posing a significant threat to patient recovery and survival. This risk of rejection can be minimized by matching the donor and host immune systems. Stem cell therapy holds significant promise for treating various disorders characterized by the loss or damage of specific cell populations. However, the potential is greatly hindered by the substantial challenge of identifying a compatible donor for transplantation. To study the effects of cell transplantation *in vivo* without the risk of rejection, immune-deprived animals are often used (Beilhack et al., 2003; Samata et al., 2015; Radaelli et al., 2015; Larsen et al., 2006; Les et al., 2018), as these are commercially available and well-*characterized* (Radaelli et al., 2018). Using immune-compromised animals for transplant modeling poses several limitations in addition to the great cost and expertise needed to work with these animals (National Research Council Committee on Immunologically Compromised Rodents, 1989). The biggest drawback is that these animals cannot provide information on how cell-based therapies might function in immune-competent recipients due to their absence of an immune system and, consequently, the immune response. Implementing embryonic stem cells (ESCs) or the generation of induced pluripotent stem cells (iPSCs) from the respective research animal is one way to address this problem (Tucker et al., 2011; Zhou et al., 2014; Lu et al., 2016; Peinkofer et al., 2021). Successful isolation and maintenance of ESCs have only been achieved for mice and not for other species (Ezashi et al., 2016). With regards to animal iPSCs, the most successful approaches for many species rely on integrative reprogramming techniques that are unable to silence the transgenes or carry the risk of reactivation after differentiation in the host (Pessôa et al., 2019). Therefore, using an immune-evasive human embryonic stem cell (hESC) line that serves as a universal donor (de Rham and Villard, 2014; Zheng et al., 2016) is a promising way for obtaining a transplantation model. Several researchers have previously published the generation of immune-evasive iPSCs and ESCs with various outcomes, demonstrating enhanced survival in different models (Frederiksen et al., 2021). A common technique to lower the immune response after transplanting cells is to create a knockout of the major histocompatibility complex (MHC) (Figueiredo et al., 2013; Wang et al., 2015; Bogomiakova et al., 2018; Norbnop et al., 2020). This will result in a lack of foreign and self-peptide presentation in the cell. Consequently, T cells cannot recognize the transplanted cells as foreign and will not initiate an immune response (York and Rock, 1996). Furthermore, it has previously been established that MHC-II knockouts are highly efficient in protecting against the presentation of peptides by antigen-presenting cells to CD4 T cells (Chen et al., 2015). One caveat is that cells that lack MHC proteins on their surface are susceptible to natural killer (NK) cell-mediated killing (Long et al., 2013). Hence, many tactics have been explored to reduce NK killing by either preserving the expression of less polymorphic MHC molecules (Taylor et al., 2012; Gornalusse et al., 2017; Han et al., 2019; Shi et al., 2020; An et al., 2022) or by overexpressing PDL-1 (Han et al., 2019) or CD47 (Deuse et al., 2019; Han et al., 2019; Deuse et al., 2021; Feng et al., 2023). Preservation of less polymorphic MHC molecules still requires immune matching, but it facilitates the identification process of a donor and can still maintain some of the favorable parts of the immune response, such as in the case of infection. However, this tactic cannot be implemented for a xenogeneic model due to the interspecies differences.

Overexpression of CD47 is known to result in a “do not eat me” signal, inhibiting phagocytosis by interacting with signal regulatory protein α (SIRPα) on macrophages (Kaur et al., 2021; Suter et al., 2021). Furthermore, it has been shown to have a protective effect against NK cells (Deuse et al., 2021). This knowledge has led to the generation of a murine and a human-induced pluripotent stem cell line with the knockout of MHC-I and MHC-II and the overexpression of CD47, both of which showed survival for at least 50 days in all tested allogenic mice and humanized mice (Deuse et al., 2019).

In this study, genomically modified hESCs were generated using CRISPR-Cas9 to knock out B2M encoding the structural part of MHC-I. The knockout was carried out by simultaneously inserting the murine version of CD47 (mCD47). Additionally, CIITA, encoding the transcription factor for MHC-II, was ablated. To monitor hESC survival *in vivo*, human-secreted embryonic alkaline phosphatase (hSEAP) was inserted and tested. Our overall goal was to test whether a knockout of MHC-I and II in combination with mCD47 overexpression is sufficient to protect hESCs from rejection in a xenogeneic setting. More specifically, our study investigated the pluripotency features and transgene expression of the generated hESCs after CRISPR-Cas9-targeted editing and tested the survival of these *in vivo* in immunocompetent BALB/c mice via the expression of hSEAP. A potential immune response and loss of transplanted cells, in addition to the hSEAP measurements, were assessed by histological staining for human stem cells and immune infiltration in the injection site.

## 2 Materials and methods

### 2.1 hESC generation

**FIGURE 1:**
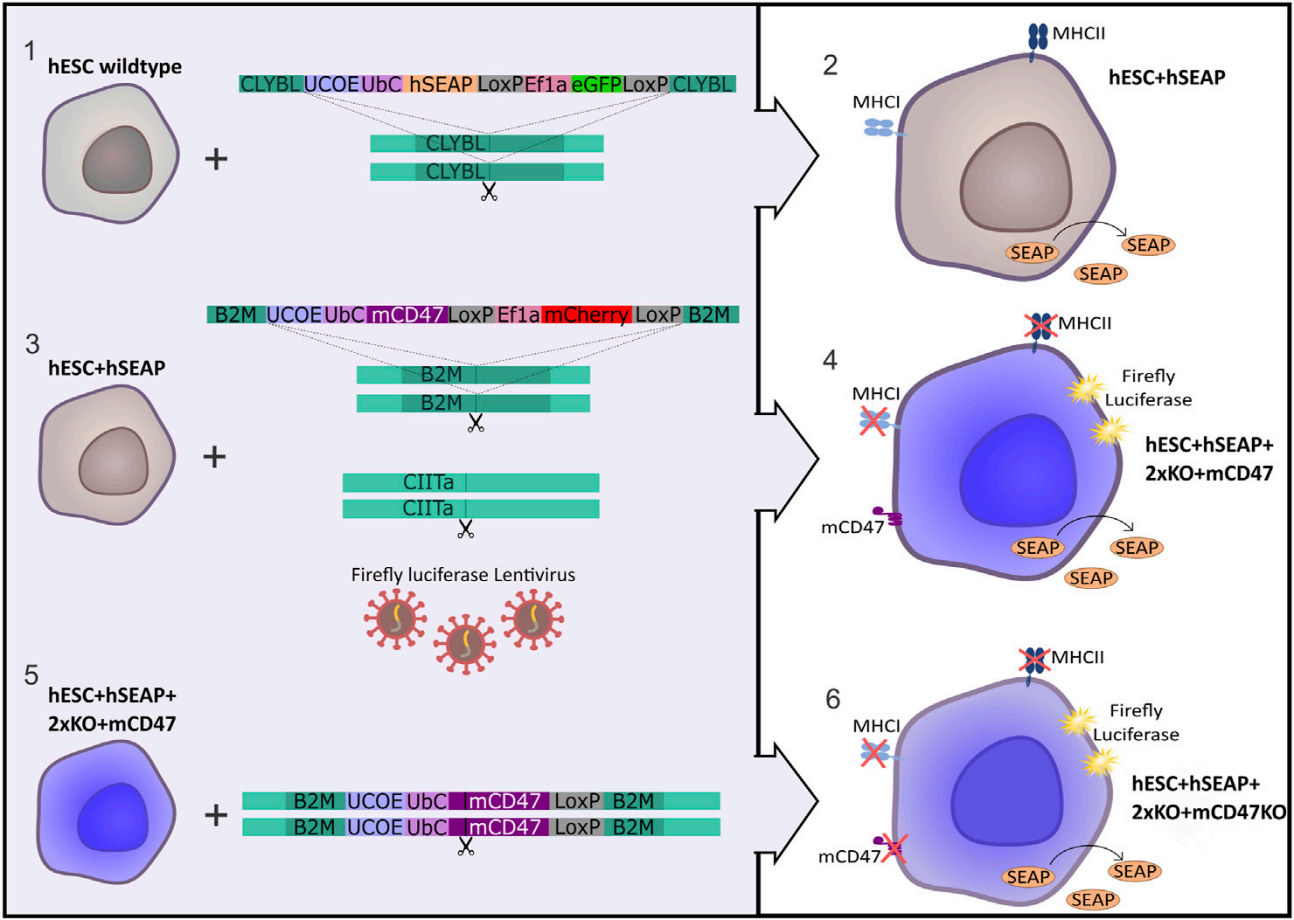
Strategy for the generation of immune-evasive human embryonic stem cells. A transgene encoding hSEAP was inserted into the safe harbor CLYBL of wildtype hESCs (1), resulting in the generation of the cell line hESC + hSEAP (2). The hESC + hSEAP had an mCD47 transgene inserted in the B2M loci, rendering B2M non-functional, and the CIITA loci targeted for knockout as well as luciferase randomly integrated by lentiviral transduction (3), which resulted in the generation of the line hESC + hSEAP+2xKO + mCD47 (4). hESC + hSEAP+2xKO + mCD47 had its mCD47 sequence targeted for knockout (5), resulting in the generation of the line hESC + hSEAP+2xKO + mCD47KO (6).

The hESC line NN GMP0050E1C3 was provided by Novo Nordisk as a research cell line and served as the foundation for the subsequent stepwise CRISPR-Cas9-mediated editing process to produce an immune-evasive hESC line. Customized plasmids used for the insertion of transgenes were ordered at GenScript (Piscataway, NJ, United States) and are illustrated in Figure 1. The complete sequence is available upon request. The design included either mCherry or GFP as the fluorescent protein tracer, which allowed for fluorescence-associated single-cell sorting of edited cells. Two LoxP sequences were placed to allow for the removal of the fluorescent protein by adding Cre-recombinase. To facilitate *in vivo* tracing, hSEAP was introduced into the CLYBL safe harbor as the initial editing step, resulting in the cell line referred to as hESC + hSEAP. The hSEAP tracer was preferred as it can be measured from blood samples, which allowed for frequent sampling without the need for anesthesia and has been published as a functional tracer *in vivo* in mice (Bao et al., 2000; Nilsson et al., 2002; Hiramatsu et al., 2005). The hESC + hSEAP line was used for all subsequent CRISPR-Cas9 editing steps.

After the confirmation of hSEAP expression *in vitro*, a new hESC line was generated by inserting splice variant 2 of mCD47 into exon 2 of the B2M loci (GRCh38 chr15:44715509-44715531), thereby disrupting the B2M sequence and creating a knockout via knock-in. Next, exon 2 of CIITA (GRCh38 chr16:10895313-10895335) was targeted with sgRNA to generate a knockout. For additional tracing, in case the hSEAP was untraceable, firefly luciferase was randomly integrated using lentivirus to allow bioluminescent imaging (Contag et al., 1997), resulting in the cell line hESC + hSEAP+2xKO + mCD47.

Lastly, mCD47 was ablated from hESC + hSEAP+2xKO + mCD47 to generate a comparative hESC line, referred to as hESC + hSEAP+2xKO + mCD47KO, to investigate the protective effects mediated exclusively by CD47.

All hESCs were screened for correct genomic insertion, the absence or presence of targeted proteins, pluripotency, the capability of differentiation into all three germ layers, and a normal karyotype. hESCs were assessed after the removal of the fluorescent cassettes by Cre-recombinase.

After the assessment, hESC + hSEAP, hESCs + hSEAP+2xKO + mCD47, and hESC + hSEAP+2xKO + mCD47KO cell lines were used for two *in vivo* pilot studies to test hSEAP as a marker to follow hESC survival *in vivo* and assess rejection.

All cells were cultured at 37°C with 5% CO_2_. Upon passage of cells, a 10 µM ROCK inhibitor (Tocris, Bristol, United Kingdom) is added to the media for the first 24 h. Reagents were bought from Thermo Fisher Scientific (Waltham, MA, United States) unless stated otherwise.

### 2.2 Gene editing

hESCs were grown on plates coated with 0.25 μL/cm^2^ iMatrix-511 (Nippi, Tokyo, Japan) in the hESC media (NutriStem hPSC XF Medium (Sartorius, Göttingen, Germany) with 0.5% human serum albumin (Akron Bio, Boca Raton, FL, United States), 0.5% penicillin/streptomycin, and 5 µM FGF (PeproTech, Cranbury, NJ, United States)). hESCs were passaged every 3–4 days with Versene (1X) and replated in the hESC media.

For editing, 0.5 µL of sgRNA (44 µM ribonucleoprotein) (IDT, Coralville, IA, United States) was mixed with 0.5 µL Cas9 (33 µM ribonucleoprotein) (IDT) and incubated for 15 min at room temperature to form the CRISPR-Cas9 complex. hESCs were harvested using TrypLE for 5 min and washed once in PBS. A total of 4*10^5^ hESCs were collected by centrifugation for 3 min at 300 G and re-suspended in 9 μL R buffer and 2 µL Cas9 electroporation enhancer (10,8 µM) (IDT). For the insertion of transgenes, plasmids were added to the CRISPR-Cas9 complex during the last centrifugation step. Re-suspended hESCs were mixed with the CRISPR-Cas9 complex and electroporated with the 10 µL Neon^TM^ Transfection System (pulse width: 20, pulse: 2, 1,100 V). hESCs were plated in the hESC media with the ROCK inhibitor and 10% KO-serum. The next day, the media was changed to the hESC media. hESCs receiving only plasmid were included as a control to assess the degradation of unintegrated plasmid. After 10 days, plasmid controls were checked for the degradation of the plasmid visible by the lack of fluorescence, and the edited cells were checked for the fluorescent signal. Subsequently, the edited cells were FACS-sorted as single cells in wells of a 96-well plate. After 14 days, the sorted hESCs were screened for the knockout of CIITA, and the hESC + hSEAP+2xKO + mCD47KO line was screened for CD47 knockout by indel detection by amplicon analysis (IDAA), which assesses the formation of indels by comparing the amplicon length of the edited cells with those of the wildtype control. hESCs were additionally screened for the insertion of hSEAP and murine CD47 by PCR amplification of the 3′ and 5′ ends of the insert, including part of the DNA backbone. Two clones from each cell line had successful editing confirmed by Sanger sequencing. Based on the sequence and growth rate, one clone from each line was selected. To determine the mono- or bi-allelic insertion, long-range PCR, covering the plasmid, homolog arms, and part of the DNA backbone, was made using the Platinum™ SuperFi II PCR Master Mix and the manufacturers’ protocol with an extension time of 3.5 min. After the assessment of plasmid insertion, the fluorescent cassettes were removed by electroporation of 100 ng Cre mRNA using the same set-up as that for editing. hESCs were single-sorted and assessed for the correct removal of the cassette by PCR amplification of the 3’ end of the plasmid. Primers used for the various PCR experiments and sequencing are listed in Table 1. All primers were ordered from Eurofins Genomics (Luxembourg).

**TABLE 1:** List of primers and oligonucleotides used in the study.

Target (analysis)	Forward primer/reverse primer (*sequencing primer*)	Size
guideRNA B2M	AAG​TCA​ACT​TCA​ATG​TCG​GA TGG (GRCh38 chr15:44715509-44715531)	
guideRNA CIITA	GCC​CCT​AGA​AGG​TGG​CTA​CC TGG (GRCh38 chr16:10895313-10895335)	
guideRNA CLYBL	TCA​CAA​GTA​CAT​CCC​CCG​GA GGG (GRCh38 chr13:99772878-99772900)	
CLYBL (IDAA)	TCT​GGA​CTA​ACC​CCA​ATC​ACG/GGT​GGG​ATT​CTT​CCT​TTC​TCT​CA	412 bp
CIITA (IDAA)	ACG​GGT​CTC​CTG​ACT​CTC​TG/GAT​GGT​GTC​TGT​GTC​GGG​TT	686 bp
B2M (IDAA)	GCG​CAA​TCT​CCA​GTG​ACA​GA/ACA​CAA​CTT​TCA​GCA​GCT​TAC​AAA	605 bp
B2M (5′ PCR)	TCG​GGT​CCA​ACT​CAA​CCA​TT/TAC​GCC​AAT​GAT​AAC​CCC​CG (GTT​GGG​AAG​GTG​GAA​GCT​CA)	1,415
B2M (3′ PCR) after Cre-Lox	GGC​CAG​ACA​TGA​TAA​GAT​ACA​TT/TGG​GAC​TCA​TTC​AGG​GTA​GTA​TG (TAC​AAG​AGA​TAG​AAA​GAC​CAG​TC)	642 bp
B2M (long-range PCR) after Cre-Lox	TCG​GGT​CCA​ACT​CAA​CCA​TT/ACC​TCC​ATG​ATG​CTG​CTT​ACA	V2: 5217 bp WT: 2226 bp
CLYBL (3′ PCR) After Cre-Lox	ATG​TTT​CAG​GTT​CAG​GGG​GAG/CAC​TCA​TTT​ACC​TAG​ACC​GGC (CCA​AAT​AGC​GGG​CAA​TGT​CAC)	533 bp
CLYBL (long-range PCR) after Cre-Lox	CTC​AGA​AGA​TGT​CAG​TAA​ACA​GTC​C/CAC​TCA​TTT​ACC​TAG​ACC​GGC​A	4764 bp WT: 1,044 bp
UCOE ddPCR	Probe: GGG​AGG​TGG​TCC​CTG​CAG​TTA​CGC​CAA​TGA​TAA​CCC​CCG​CCA​GAA​AAA​TCT​TAG​TAG​CCT​TCC​CTT​TTT​GTT​TTC​CGT​GCC​CCA​ACT​CGG​CGG​ATT​GAC​TCG​GCC​CCT​TCC​GGA​AAC​ACC​CGA	
Off-target CLYBL Chr6	GTG​TTC​AAG​TAA​GTA​ACC​CCC/CAT​CTC​ATC​TCC​TAT​CTC​TCC​C (AGT​GCA​AGA​GTA​GCG​ATG​CTG​AC)	312 bp
Off-target CLYBL Chr5	GTC​TCC​AAA​GTT​ACC​AGA​AAC​C/GTC​CCC​TAA​AGT​CCA​AAA​GTG (GAG​CAC​CTG​TCA​AAG​TTC​TAT​AGC)	407 bp
Off-target CLYBL CHr16	CCC​ACT​AGG​ACA​TCA​CAC​CC/CAT​CAT​CGC​CCC​TTG​GTG​AC (CTC​CAT​CTA​AAG​GCG​CTG​CTT​G)	367 bp
Off-target CLYBL Chr7	ACC​TGA​AGT​CGG​GGG​TAG​AG/AAC​CCC​TCC​TGT​CTC​CCA​TT (GCA​GGT​GCT​CCA​TAG​GAA​GG)	377 bp
Off-target CLYBL Chr10	CCT​CTC​AAG​TCC​AGT​GTG​GC/CCT​CAC​ACT​TTT​GCT​TTC​CCG (TGT TTG AAA GGT AGA CAG TGT G)	494 bp
Off-target CIITA Chr11	CCT​CCT​CCT​TCA​CTT​CTT​CC/CAT​TTT​CCA​ACA​TGA​CAC​ACC (TGC​CTC​AGG​GTC​TTT​GCA​CTT​ATG)	796 bp
Off-target CIITA Chr17	CTG​TTC​AGT​GAG​CCT​GGT​CC/ATG​TCC​TGG​GGT​CTG​ACT​CT (AGC​CAC​CTC​AGA​GGA​GCA​AAC​ATC)	580 bp
Off-target CIITA Chr3	GGC​TAT​CTA​CTC​TGC​CCG​AC/TGC​ATA​TTC​ATG​AAC​GCG​GG (TCC​TCT​CCG​CCC​AGA​TAT​CAG​TTC)	462 bp
Off-target CIITA Chr16	TAG​GGA​ACA​ACG​AGC​GAA​CC/GCG​ACA​AGA​GCT​CTA​CCT​GG (AAC​AAA​GCC​TTC​TGT​CTG​CC)	630 bp
Off-target CIITA Chr19	CTC​AGG​ACC​CTG​CAG​ATG​AC/ACC​CGA​GCT​GAG​TGT​CTA​GG (GGG​AGA​GGC​CAG​AAA​CGA​ACT​ATG)	698 bp
B2M off-target Chr3	GTT​GGG​GCA​TAG​AGA​ACC​CC/ACG​CAA​CCT​GAG​TCA​ATA​GCA (TTC​CGA​AGA​ATA​AAA​ATG​GAA​A)	697 bp
B2M off-target Chr5	CCA​GAT​GCC​TGC​AGA​GTT​GA/GAA​GCC​AGT​TGC​AAA​CCC​AG (AGC​TTT​TGA​ACT​CTT​CAG​AGT​AAG​C)	635 bp
B2M off-target Chr6	CTT​GTG​GCT​TCT​GGG​TGA​CA/AAA​AGA​GCT​GAC​GCA​AAG​CAC (ACT​ACA​GCC​AGA​GTG​GGG​TG)	518 bp
B2M Off-target Chr8	TGA​CTG​ACT​CAT​GCC​ATC​TTG/GCC​ATT​AAT​AAA​ACT​GCT​GCA​CA (TTG​CTT​CAT​CTT​CAG​AGA​CTA​GTG​G)	482 bp
B2M off-target Chr17	TAC​AAA​GCA​CGC​TGG​CTA​CA/GGC​AGC​TGT​AAG​CGT​ATT​CC (TAT​TTA​TCC​CAT​GCC​ATT​CTT​TTT)	752 bp
CD47 deletion	GTC​GTT​GAA​ACA​AGG​TGG​GG/TCA​CTG​CAT​TCT​AGT​TGT​GGT	1,614 bp With deletion: 407 bp
cDNA CD47 BALB/c	TGG​TCA​TCC​CTT​GCA​TCG​TC/TGA​AAT​CAA​AAG​GGG​GCC​G (CAC​CGA​AGA​AAT​GTT​TGT​GAA​G)	767

V2, hESC + hSEAP+2xKO + mCD47; WT, wildtype.

### 2.3 Flow cytometry

hESCs were harvested with Versene for 15 min and differentiated cells with Accutase for 5 min, washed with PBS, and either fixated and permeabilized using the transcription factor buffer set from BD Bioscience (cat# 562574) before staining or directly stained by resuspension in flow buffer (PBS + 1% BSA (Sigma)) + antibody (Table 2). The cells were incubated with antibodies for 15–30 min at 4°C, followed by 3 min of centrifugation at 300 G. Subsequently, the cells were washed twice in flow buffer and finally re-suspended in 200 µL flow buffer. The cells were analyzed on the cytoFLEX S (Beckman Coulter, Brea, CA, United States), and the data were processed in FlowJo.

**TABLE 2:** Antibodies used for flow cytometry, immunocytochemistry, and immunohistochemistry.

Antibody	Dilution	Vendor category #
*Pluripotency marker*		
Mouse anti-OCT4	1:200	Santa Cruz Cat# SC-5279
Rabbit anti-NANOG	1:50	PeproTech Cat# 500-P236
Rat anti-SSEA3	1:100	BioLegend Cat# 330302
Mouse anti-SOX2-V450	1:50	BD Biosciences Cat#561610
Mouse anti-Oct3/4-AF647	1:15	BD Biosciences Cat#560329
*Differentiation marker*		
Rabbit anti-alpha-1-fetoprotein	1:200	DAKO Cat# A0008
Mouse anti-smooth muscle actin	1:100	DAKO Cat# M0851
Mouse anti-beta-III-tubulin	1:200	Sigma-Aldrich Cat# T8660
Mouse anti-SOX2-BV421	1:130	BioLegend Cat# 656114
Human recombinant OTX2	1:320	Miltenyi Cat# 130-121-193
Human recombinant PAX6	1:160	Miltenyi Cat# 130-123-267
Mouse anti-Ki67-AF488	1:2500	BD Bioscience Cat# 561165
*CD47 marker*		
Rat anti-mouse CD47-PE-Cyanine7 Monoclonal Antibody (miap301)	1:40	Thermo Fisher Scientific Cat# 25-0471-80
Rat anti-mouse CD47^−^ FITC, Monoclonal Antibody (miap301)	1:40	Thermo Fisher Scientific Cat# 11-0471-82
Rat IgG2a kappa Isotype Control (eBR2a), FITC	1:40	Thermo Fisher Scientific Cat# 11-4321-80
Rat IgG2a kappa Isotype Control (eBR2a), PE-Cyanine7	1:40	Thermo Fisher Scientific Cat# 25-4321-81
PE-Labeled Mouse SIRP alpha Protein, His Tag	1:25	ACROBiosystems Cat# SIA-MP2H6-25 tests
*Marker for knockout assessment*		
FITC anti-human HLA-A, B, C W6/32	1:20	Nordic Biosite Cat# 311403
FITC Mouse IgG2a, κ Isotype Ctrl	1:20	Nordic Biosite Cat# 400207
*Secondary antibody*		
Nuclear stain DAPI	1:1,000	BD Biosciences Cat# 564907
Donkey anti-Rabbit IgG Alexa Fluor 488	1:200	Life technologies Cat# A21206
Donkey anti-Mouse IgG Alexa Fluor 594	1:200	Thermo Fisher Scientific Cat# A-21203
Donkey anti-Rat IgG Alexa Fluor 488	1:200	Thermo Fisher Scientific Cat# A-21208
Donkey anti-rabbit IgG-Cy3	1:100	Jackson Cat#711-165-152
TSA-Cy3	1:100	Perkin Elmer Cat# SAT704B001 EA
Anti-rabbit HQ	Ready to use	Roche Cat# 760-4815
Anti-HQ-HRP	Ready to use	Roche Cat# 760-4820
Donkey anti-rabbit_biotin	1:200	Jackson Cat# 711-065-152
Streptavidin	1:500	Perkin Elmer Cat# 004303
Rb-Brightvision_HRP	Ready to use	Immunologic Cat# VWRKDPVR110HRP
Rhodamine kit	Ready to use	Ventana Cat# 760-233
*Immunohistochemical marker*		
Rabbit anti-KU80	1:300	Cell Signaling Cat# 2180s
Rabbit anti-CD45	1:1,500	Abcam Cat# Ab10558
Rabbit anti-CD3e (SP7)	1:200	Thermo Fisher Scientific Cat# RM-9107-S1
Mayer’s hematoxylin solution	Ready to use	Sigma Cat# MSH80-2.5L
Eosin Y solution	1:100	Sigma Cat# HT110280-2.5L

### 2.4 Immunocytochemistry

hESCs were washed with PBS, after which they were fixated in 4% PFA for 15 min. The fixated hESCs were washed three times in PBS before permeabilizing with 0.2% Triton X (Sigma) for 20 min, followed by 30 min of blocking with 3% BSA. Primary antibody (Table 2) was diluted in 3% BSA and added and incubated O/N at 4°C. The next day, hESCs were washed three times, and the secondary antibody was added for 1 h RT in the dark. hESCs were washed three times, after which DAPI (Table 2) was added for 7 min in the dark. Finally, hESCs were washed four times, PBS was added to the well, and images were obtained on the same day using a fluorescent microscope. The plates were stored in the dark at 4°C until use.

### 2.5 Spontaneous differentiation and neural differentiation

Spontaneous differentiation was achieved by the formation of embryoid bodies for 7 days. hESCs were collected in colonies by Accutase treatment for 1 min, cell scraping, and 1 min centrifugation of the collected cells. hESC clusters were carefully re-suspended in hESC media and plated in low-attachment plates with media change every second day. After 7 days, embryoid bodies were transferred to Matrigel-covered plates with fibroblast media (DMEM+10%FBS + FGF + P/S). The media was changed every second day. After 14 days, the cells were immunocytochemically stained for the trilineage markers alpha-fetoprotein (AFP), smooth muscle actin (SMA), and beta-III tubulin (TUBIII) (Table 2).

To investigate the presence of promoter shutdown, hESCs were differentiated into forebrain neural precursors following a protocol adapted from Gantner et al., 2021. Before differentiation, hESCs were adapted to iPS-brew XF basal media (Miltenyi Biotec, Bergisch Gladbach, Germany) and laminin-521 (BioLamina, Sundbyberg, Sweden) coating. On day −1, hESCs were split with Accutase and plated as a confluent monolayer. The next day, hESCs were washed with PBS, and the media was changed to neural media (96% 1:1 DMEM and Neurobasal, 2% B27, 1% N2, 1% NNEA, 0.5% P/S, 0.5% GlutaMAX, and 0.09% β-mercaptoethanol) with the addition of SMAD inhibitors (100 nM LDN193189 and 10 µM SB431542) to induce neuroectodermal induction. Media was changed daily, and after 11 days, cells were passaged 1:2.5 with EDTA. The next day, the media was changed to neural media with 20 ng/mL FGF, with daily media change until day 18. On day 18, cells were passaged with Accutase and seeded at a 1:5 ratio. The media was changed the following day to neural media with FGF2. On day 21, the media was changed to neural media supplemented with 200 nM ascorbic acid, 40 ng/mL BDNF, 40 ng/mL GDNF, 50 µM dcAMP, and 1 μg/mL mouse laminin. After this, the media was changed every second day until day 35, at which hSEAP expression was assessed. On day 18, some of the cells were harvested and analyzed for neural differentiation markers and mCD47 expression (Table 2).

### 2.6 hSEAP assay

The amount of hSEAP in the cell media and serum was measured using the kit and the protocol from the Phospha-Light™ SEAP Reporter Gene Assay System. In short, 50 μL of cell media was diluted with a 50 µL dilution buffer and heated for 30 min at 65°C. The diluted sample (50 µL) was incubated at RT with a 50 µL assay buffer, after which a 50 µL reaction buffer was added for 20 min. The reaction was analyzed in a black-well 96-well plate using a luminometer for 0.1 s.

For serum samples, 12–25 µL of serum was mixed with 38–25 µL of the dilution buffer and processed as described above.

### 2.7 Animal model

**FIGURE 2:**
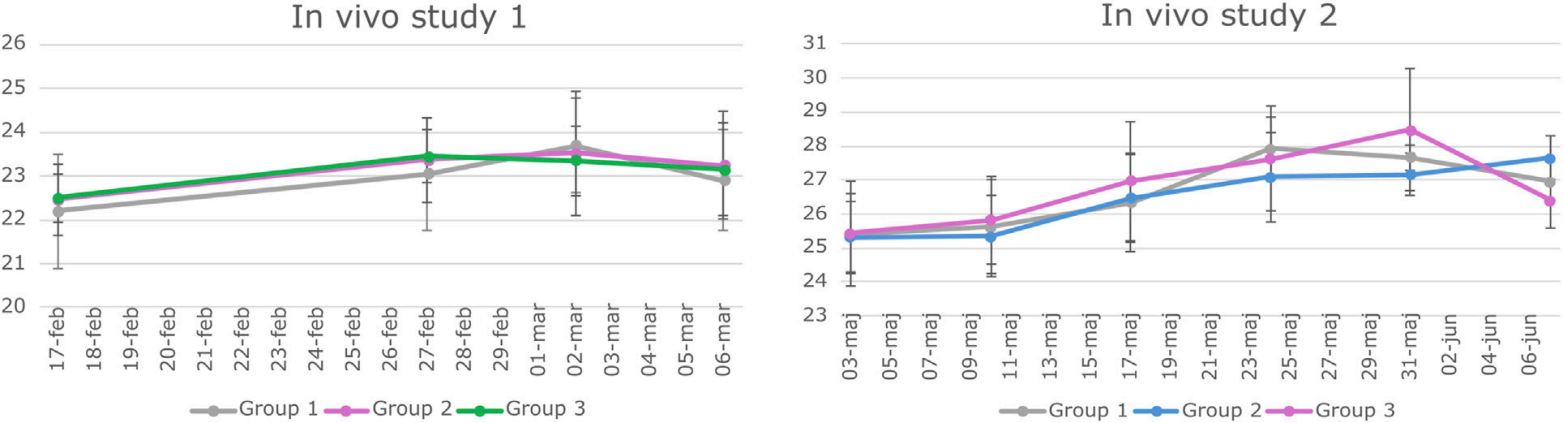
Weight data of animals involved in study *1* and Study 2. BALB/c mice underwent weekly weighing throughout both in vivo studies. The graphs depict the mean weight of each experimental group along with the standard deviation (SD) at various time points. Statistical analysis using a two-way repeated measures ANOVA indicated no significant difference in weight among the groups over time.

The *in vivo* studies were conducted according to the European legislation on animal experimentation (directive 2010/63/EU) and were approved by the Danish Animal Experiment Inspectorate and the Novo Nordisk Animal Welfare Council. All the studies were reported according to the ARRIVE guidelines (Percie du Sert et al., 2020). BALB/c mice were housed in groups of 4–6 animals per cage under the standard conditions (12/12 h light–dark cycle with *ad libitum* access to standard chow and water). Animals were acclimatized for 11 days, after which they were weighed once a week (Figure 2) and observed daily with a focus on the size of the subcutaneous transplant.

For the *in vivo* studies, edited hESCs had been single-cell sorted based on the presence of the fluorescent molecules GFP or mCherry expressed by the insert (Supplementary Figure S2). Single-sorted cells were expanded, genotyped, and sequenced as previously described to ensure the correct insertion and a pure population of edited cells. hESCs were collected by Versene for 15 min and re-suspended in 100 µL of ice-cold Matrigel, kept in separate marked tubes, and stored on ice until transplantation. Transplantations were performed on anesthetized mice. Anesthesia was induced in an induction chamber (4% isoflurane and 1L/min O_2_) and maintained through a nose cone (2% isoflurane and 1L/min O_2_). The injection site was shaved and cleaned with ethanol, and the negative toe-pinch reflex was verified before initiating injection.

hESCs in Matrigel were mixed once by pipetting and collected using a G30 insulin needle. All equipment was kept on ice to prevent the solidification of the Matrigel. Using the insulin needle, hESCs were subcutaneously injected into the left dorsal flank region.

During the studies, blood samples were obtained to follow the survival of the graft. At each time point, approximately 100 µL of blood was sampled from the sublingual vein into a 100 µL lithium-heparinized Microvette (Sarstedt, Nümbrecht, Germany). Blood was centrifuged for 10 min at 1.500 x g at 4°C to collect serum. Serum was transferred to freezing tubes placed on dry ice and stored at −80°C.

For the collection of tissue, the animals were terminally anesthetized in an induction chamber (4%–5% isoflurane, 1L/min O2), and up to 1 mL of intra-orbital blood was obtained and processed as described above. After blood sampling, the animals were euthanized by cervical dislocation, and various tissue samples were collected: the injection site was dissected from the surrounding tissue, and the regional lymph node draining the implantation site was macroscopically evaluated and collected. Both were placed in 4% PFA. Additionally, the spleen, kidney, and liver were dissected, placed in 4% PFA, and kept for potential future studies. Injections and euthanasia were conducted in a blinded manner by the person carrying out the procedures. No animals were excluded from the studies, and no signs of distress or pain were observed. Humane endpoints included general humane endpoints as well as any signs that the graft size affected normal body function.

### 2.8 *In vivo* study I

**FIGURE 3:**
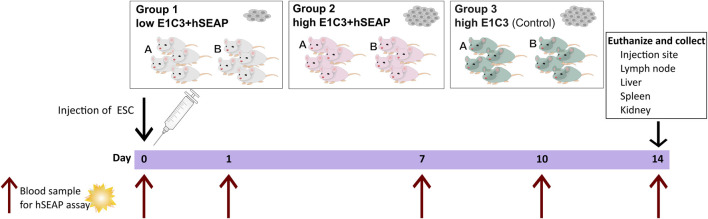
Schedule of in vivo study I. On day 0, animals were subcutaneously injected with either a low number of hESC + hSEAP, a high number of hESC + hSEAP, or wildtype hESCs. To assess hESC survival, blood samples were taken on days 0, 1, 7, 10, and 14 and analyzed for the presence of hSEAP (maximum one blood sample per animal per week). On day 14, the animals were euthanized, and tissue was collected and stored in 4% PFA.

Study I was a pilot study designed to test if the inserted hSEAP could be monitored *in vivo* and provide information on the survival of immunogenic hESCs (cell line E1C3) in immunocompetent mice (Figure 3). For this, 24 male 10-week-old BALB/c mice with an average weight of 22.4 ± 0.9 g (mean ± SD) from Janvier Labs (France) were divided into three groups (N = 8) and stratified by weight. Group 1 received subcutaneous transplantation of low-dose (1*10^5^) hESC + hSEAP. Group 2 received subcutaneous transplantation of high-dose (2.5*10^6^) hESC + hSEAP, and group 3 received subcutaneous transplantation of high-dose (2.5*10^6^) non-edited hESCs.

To uphold animal welfare legislation and not exceed the recommendations of blood sample volume per animal during the course of the study, animals from each group were sub-grouped into groups A and B, respectively. Animals from group A were sampled on day 0 before anesthesia and on day 7. Group B was sampled on days 1 and 10.

On day 14, the animals were euthanized, and the study was terminated.

### 2.9 *In vivo* study II

The second *in vivo* study was designed as a pilot study to test whether the knockout of B2M and CIITA in combination with mCD47 overexpression could protect transplanted hESCs from rejection in an immunocompetent host. To assess the effect of mCD47, a mCD47 knockout cell line hESC + hSEAP+2xKO + mCD47KO was included.

**FIGURE 4:**
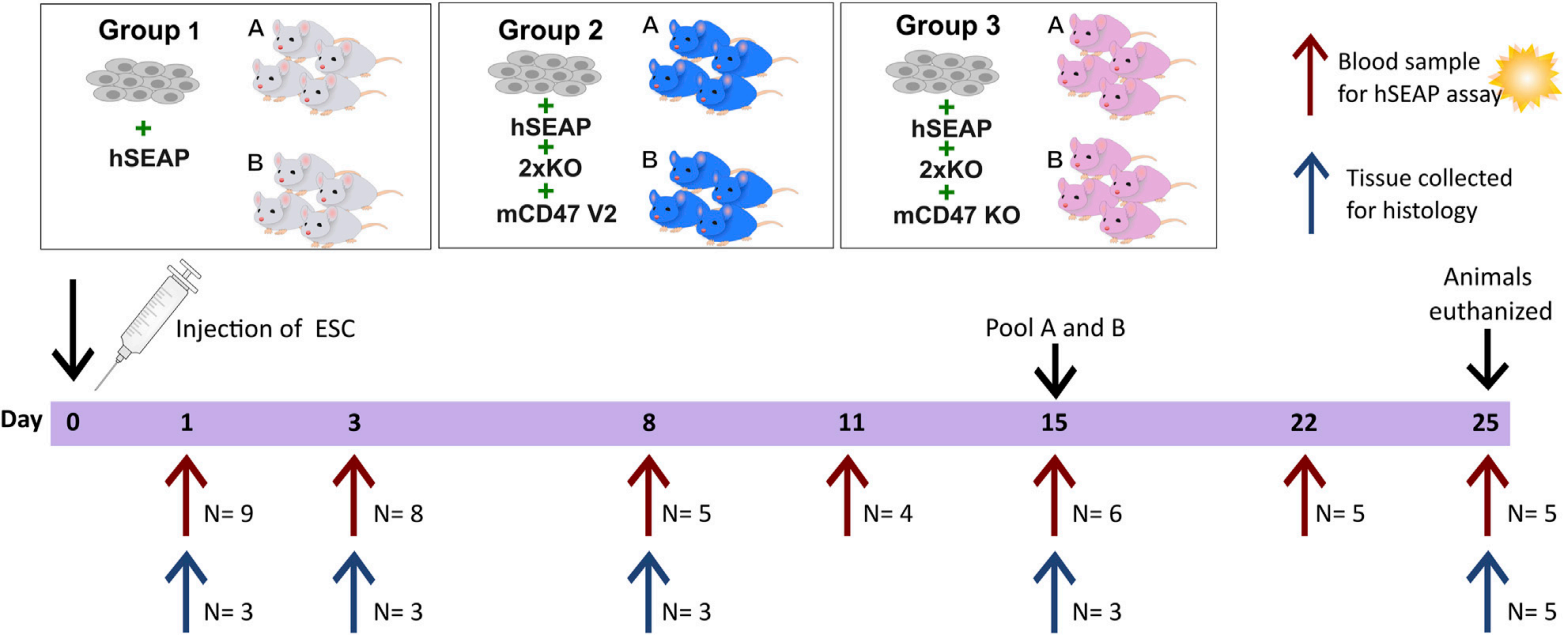
Schedule of in vivo study II. On day 0, animals were randomly divided into three groups and injected with either hESCs expressing hSEAP (control), hESC + hSEAP+2xKO + mCD47 V2, or hESC + hSEAP+2xKO + mCD47 V2 KO. Animals were subdivided into groups a and b for blood sampling, which was taken on days 1, 3, 8, 11, 15, 22, and 25 (maximum one blood sample per animal per week). Serum was extracted from blood and analyzed for hSEAP. On days 1, 3, 8, 15, and 25, a subset of animals were euthanized, and tissue was collected for the histological assessment of hESC survival and immune response.

The design of study II was based on findings from study I. Using the hSEAP means from day 1 of study I, a power of 0.8, and an alpha value of 0.05, the group size for blood sampling was calculated to be *n* = 4 (Figure 4). The sample size for the collection of tissue for histology was set to three, as the study set out to confirm the findings from the hSEAP measurement and give an indication for immune response by either showing the presence or lack of stained cells (binomial endpoint).

Eight-week-old male BALB/c mice with an average weight of 25.5 ± 1.3 g (mean ± SD) from Charles River (Germany) were randomized into three groups stratified by weight (N = 18) using a computer-based random number generator. All groups had 1.25*10^6^ hESCs subcutaneously transplanted. Group 1 received hESC + hSEAP, group 2 received hESC + hSEAP+2xKO + mCD47, and group 3 received hESC + hSEAP+2xKO + mCD47KO.

Animals from the three groups were randomly sub-grouped into a and b for blood sampling. Blood samples from sub-group a were collected on days 1, 8, and 15. Blood samples from sub-group b were collected on days 3 and 11. On day 22, samples from groups a and b were pooled for the remaining study blood collected. To determine hESC survival during the study, serum was extracted, as illustrated in Figure 4, and analyzed within 24 h of collection.

To assess the injection site at different time points, three animals were euthanized from each of the three groups on days 1, 3, 8, and 15, as described above. For days 1, 3, and 8, a non-treated animal was euthanized and included as a control. On day 25, the 5 remaining animals in each group were euthanized, and tissue and blood were collected as previously described.

One animal had a bite mark in the injection area, which could be signs of itch and irritation or a bite from another mouse.

### 2.10 Histology

The tissue was fixed in 4% PFA for at least 72 h, after which the injection site was divided into halves. Both halves of the implantation site and the regional lymph node were dehydrated in ethanol and cleared in Clearene Solvent (Leica, Wetzlar, Germany), after which they were embedded in paraffin. The blocks were sectioned into 3–5 µm slices and arranged on glass slides, with each slide containing a section each from the halves of the injection site and the lymph node.

The slides were H&E stained by removing paraffin with 3 × 5 min xylene treatment, followed by 3 × 5 min 99% ethanol, 2 × 5 min 96% ethanol, 1 × 5 min 70% ethanol, and 5 min deionized H_2_O treatment. Afterward, the slides were treated with Mayer’s hematoxylin (Table 2) for 3 min and washed in tap water for 5 min. The slides were stained with 0.1% eosin (Table 2) for 1 min, shortly washed in deionized H_2_O, and dehydrated in increasing concentrations of ethanol (70%–99%).

The slides were fluorescently stained after the removal of paraffin, as described above. The slides were treated with TEG and microwaved for 15 min, followed by 5 min of rinsing in tap water. Then, 1% H_2_O_2_ was added for 15 min and washed for 2 min in tap water. To reduce non-specific binding, the slides were treated with 0.05% Tween 20 in TBS for 3 × 2 min and blocked with 0.5% TNB buffer for 30 min. Primary antibodies were diluted in TNB according to Table 2 and incubated for 1 h at room temperature or overnight at 4°C. Afterward, the slides were washed for 3 × 2 min in 0.05% Tween 20 and treated with the secondary antibody (Table 2) and DAPI for 30 min at RT, followed by 3 × 2 min wash, after which fluorescent dye was diluted (Table 2) and added for 15–30 min. Lastly, the slides were washed in 0.05% Tween 20 and rinsed in deionized H_2_O, after which the slides were mounted in fluorescent mounting. The quantification of fluorescent signals was blinded and carried out using ImageJ software.

## 3 Results

### 3.1 Assessment of hESCs upon editing

**FIGURE 5:**
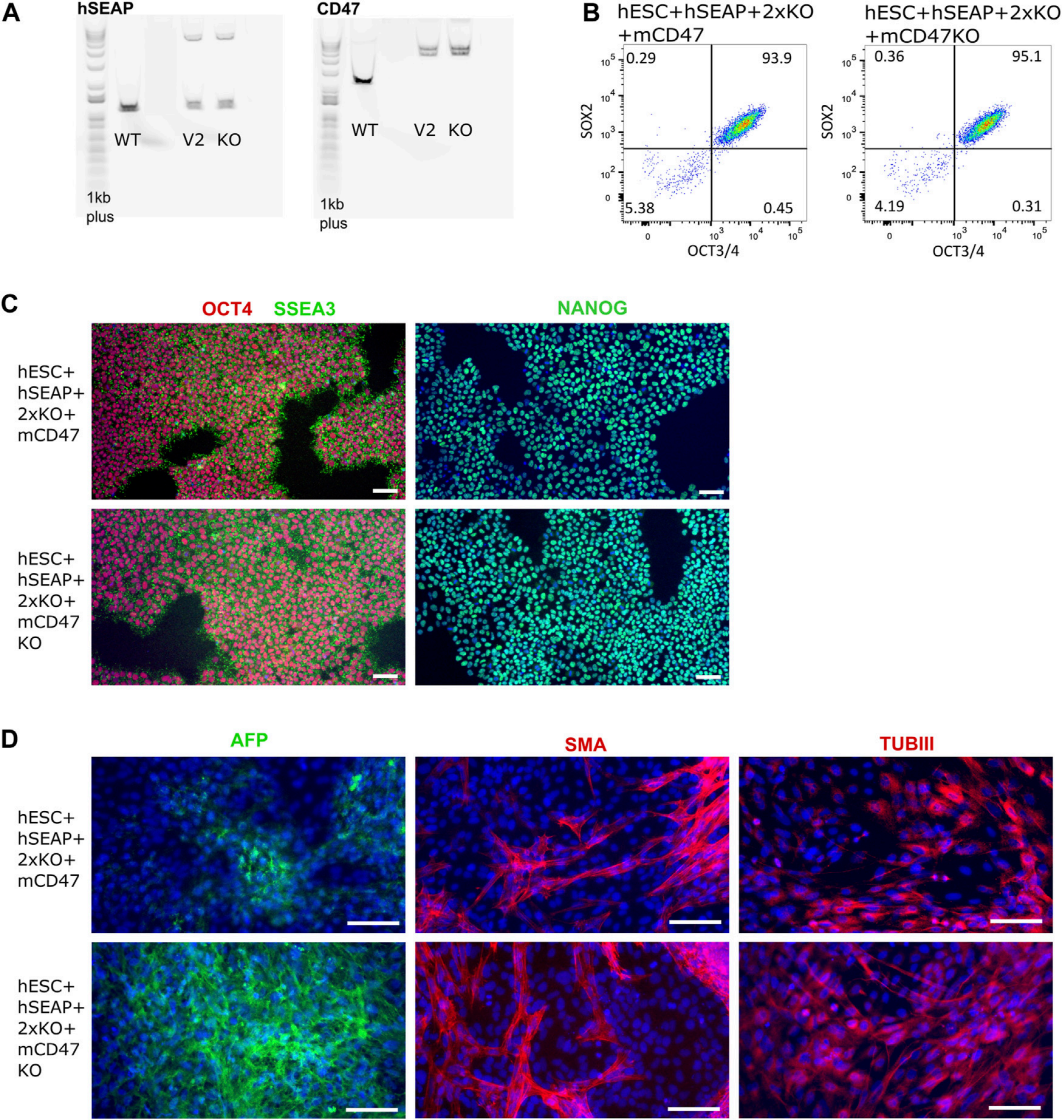
Insertion of CD47 and hSEAP and pluripotency profiles of the resulting hESC lines. **(A)** Long-range PCR amplicon covering the site of insertion in B2M (CD47) and CLYBL (hSEAP). WT, wildtype hSECs with no transgene; V2, hESC + hSEAP+2xKO + mCD47; and KO, hESC + hSEAP+2xKO + mCD47KO. Insertion of hSEAP produces a 4,764 bp product, and CD47 insertion results in a 5,217 bp product. 1Kb plus DNA ladder was used. **(B)** Flow cytometry data showing the percentage of hESCs positive for OCT4 and SOX2 in the edited hESCs. **(C)** Immunocytochemical staining for pluripotency markers OCT4, SSEA3, and NANOG on gene-edited hESCs. Scale bars= 100 µm. **(D)** Immunocytochemical staining of the gene-edited hESCs after 3 weeks of spontaneous differentiation. Markers for each of the three germ layers were used to assess differentiation potential. Mesoderm= smooth muscle actin (SMA), ectoderm = beta tubulin 3 (TUBIII), and endoderm= AFP. Scale bars= 100 µm.

The guides (sgRNAs) used in this study have previously been shown by IDAA to have a cutting efficiency above 50% (Supplementary Figure S1). For the insertion of transgenes, the length of the homology arms gave varying integration efficiencies. Insertion of the transgene encoding hSEAP with 300 bp arms showed ∼1% integration efficiency, and the transgene encoding for mCD47 with 800 bp arms showed integration efficiency of 0.4–3.8% (Supplementary Figure S2). Correct insertion of the transgenes was confirmed by long-range PCR, which showed mono-allelic insertion of hSEAP and bi-allelic insertion of mCD47 (Figure 5A). Digital droplet PCR confirmed the mono- and bi-allelic insertion and showed no random integration of the transgenes (Supplementary Figure S3). The selected edited hESCs showed the expression of the pluripotency markers OCT4, NANOG, and SSEA3 by immunocytochemical staining (Figure 5B), and more than 93% of the hESCs stained positive for OCT4 and SOX2 in flow analysis (Figure 5C). Pluripotency was further confirmed by the hESCs’ abilities to differentiate into cell types from all three germ layers upon spontaneous differentiation (Figure 5D). All the results were comparable to the pluripotency state of the non-edited wildtype hESCs (Supplementary Figure S4).

Several other tests were conducted that confirmed the quality of the hESCs after editing, including PluriTest, KaryoStatTM, STR, and off-target sequencing (Supplementary Figures S5–S8).

### 3.2 Transgene expression

**FIGURE 6:**
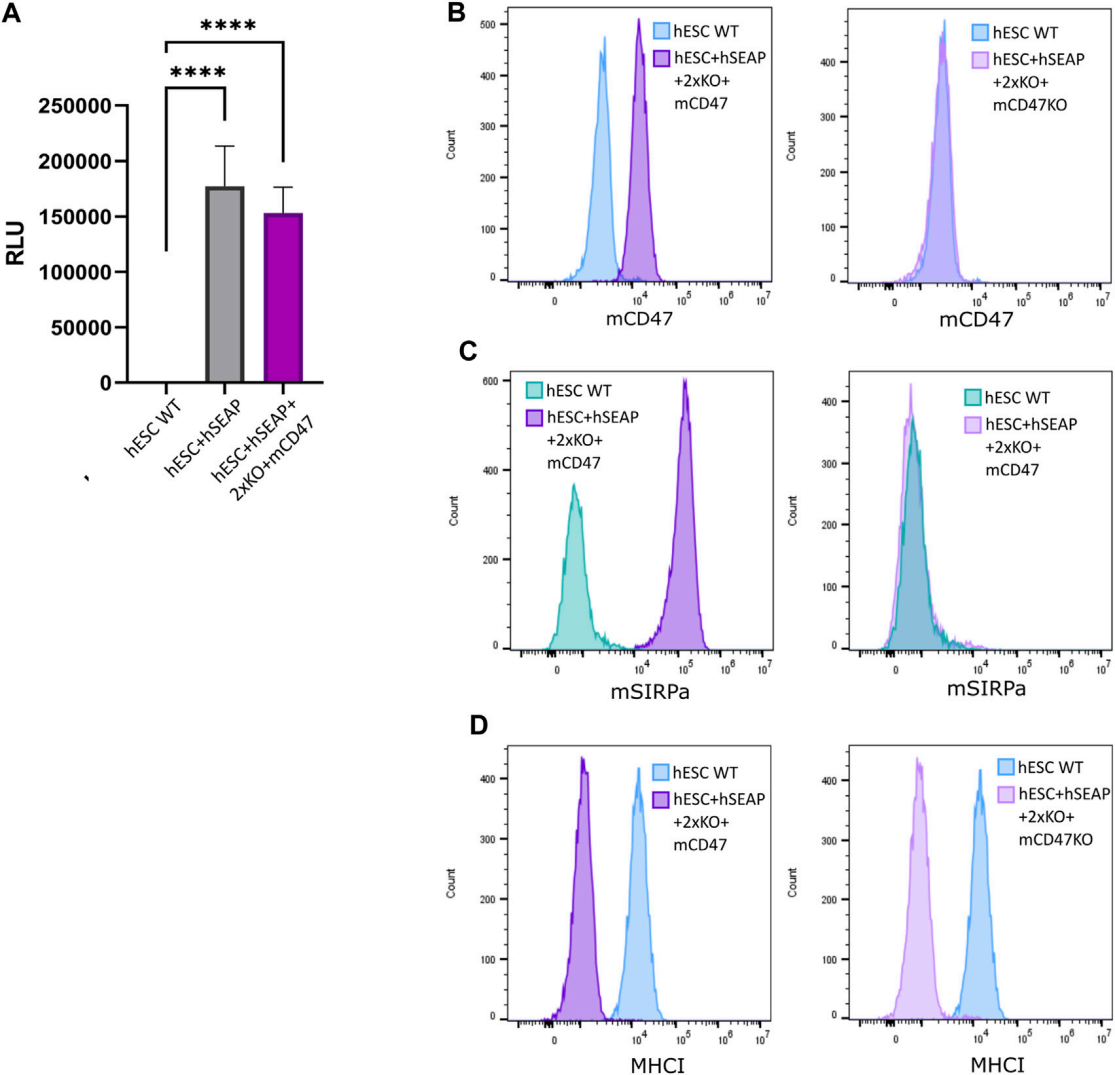
Expression of hSEAP, mCD47, and MHC-I on gene-edited hESCs. **(A)** In vitro detection of hSEAP secreted to cell media by gene-edited hESCs. hSEAP is measured by chemiluminescence and compared to wildtype hESCs by one-way ANOVA **** = *p* < 0.001. **(B)** Detection of murine CD47 expression measured by flow cytometry on the generated hESCs and compared to the expression in wildtype hESCs. **(C)** Binding of murine CD47 to murine SIRPa was assessed by the incubation of fluorescently labeled recombinant murine SIRPa with generated hESCs and wildtype hESCs as the control, followed by flow analysis. **(D)** Expression of HLA-A, B, and C measured by flow cytometry for generated hESCs and compared to wildtype hESCs.

The expression of the inserted hSEAP was assessed *in vitro* and showed a significantly increased signal compared to wildtype hESCs (Figure 6A). Additionally, luciferase expression was assessed and showed luminescence upon treatment with luciferin (Supplementary Figure S9). Murine CD47 was detected by flow analysis in hESC + hSEAP+2xKO + mCD47 but could not be detected in the knockout line hESC + hSEAP+2xKO + mCD47KO (Figure 6B). The functionality of the expressed mCD47 was demonstrated by a binding assay to murine SIRPα, in which 100% binding was seen for hESC + hSEAP+2xKO + mCD47, but no binding was seen for the knockout line hESC + hSEAP+2xKO + mCD47KO (Figure 6C). These results strongly indicate that the inserted mCD47 transgene in hESC + hSEAP+2xKO + mCD47 can mediate an anti-phagocytic signal by interacting with the SIRPα receptor, which could not be seen for hESC + hSEAP+2xKO + mCD47KO. Flow cytometry for MHC-I revealed no expression, which confirmed the knockout because of the transgene insertion in B2M (Figure 6D). Sequencing of the target site in CIITA showed the generation of frameshift (Supplementary Figure S10), disrupting the MHC-II expression.

Sequencing of the 5’-end of the transgenes showed correct removal of the fluorescent cassette for both hSEAP and mCD47 (Supplementary Figure S11). For mCD47, sgRNA cut 81 bp downstream from where the homology arms were designed. This results in an 81 bp deletion in the 5′ and an 81 bp insertion in the 3’ end. As the study aimed to knock out the *B2M* gene, the 81 bp swap is of no consequence and may increase the chance of an efficient knockout.

The full length of the inserted mCD47 transgene was sequenced to check for unexpected genomic alterations. As both hESC-hSEAP+2xKO + mCD47 and hESC-hSEAP+2xKO + mCD47KO reside in the same clone transfected with the mCD47 transgene, the sequencing results are identical, except for the knockout in hESC-hSEAP+2xKO + mCD47KO (Supplementary Figure S12). In addition to the 81 bp shift from the homology arms previously described, a heterozygous deletion from 2,709 bp to 3,916 bp was seen, covering the last part of the UbC promoter and the first half of the CD47 coding region (Supplementary Figure S12). This deletion causes mono-allelic expression of mCD47, despite a bi-allelic insertion. To investigate whether the deletion was seen due to folding of the genomic DNA used for PCR, increased denaturing temperature, time, and GC-enhancer were applied in the PCR, but it showed no change in amplicon, indicating the hairpin formation of the plasmid during editing (Supplementary Figure S13).

### 3.3 Transgene expression upon differentiation

**FIGURE 7:**
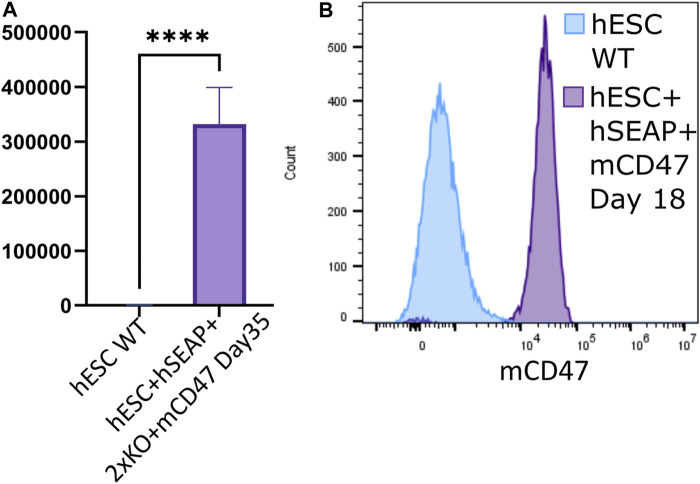
hSEAP and mCD47 expression in gene-edited hESCs after directed neural differentiation. **(A)** Expression of hSEAP in gene-edited hESCs after 35 days of neural differentiation. Comparison with differentiated wildtype hESCs using Student’s t-test **** = *p* < 0.0001. **(B)** mCD47 expression by flow cytometry for gene-edited hESCs and wildtype hESCs after 18 days of neural differentiation.

Through differentiation using a neural differentiation protocol, hESC + hSEAp+2xKO + mCD47 showed the expression of neural markers (Supplementary Figure S14) and continued to show significant expression of hSEAP (Figure 7A) and mCD47 (Figure 7B) *in vitro*, which supports the notion that no promoter shutdown is taking place as a result of the differentiation. As hESC + hSEAP and hESC + hSEAP+2xKO + mCD47KO carry the identical hSEAP transgene as hESC + hSEAp+2xKO + mCD47, the lack of promotor shutdown in this line is expected to be representative for all the lines. Since both hSEAP and mCD47 were expressed continuously, the generated hESCs were further tested in an *in vivo* setting.

### 3.4 *In vivo* assessment of transplanted hESCs

**FIGURE 8:**
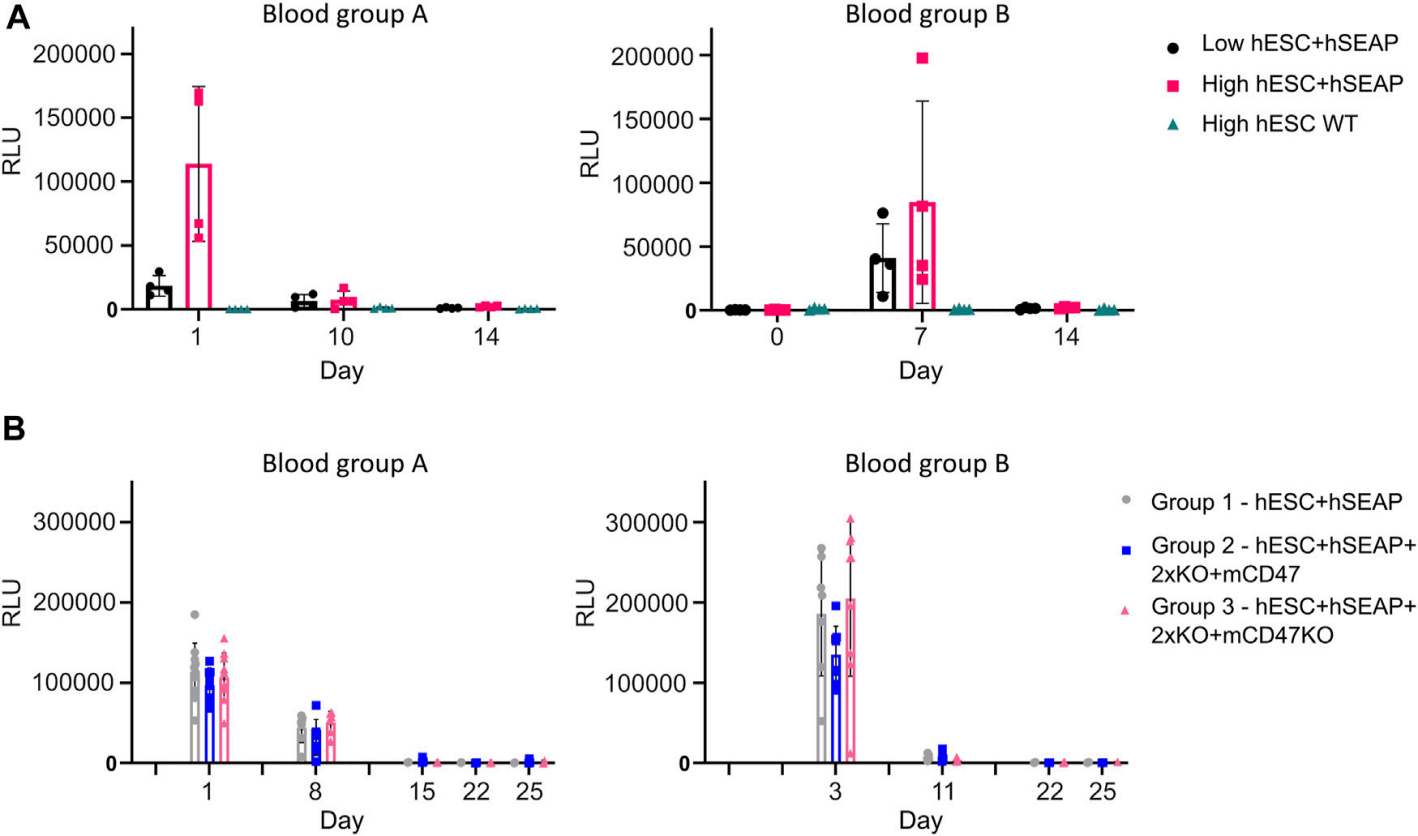
hSEAP in serum samples from in vivo studies I and II. **(A)** hSEAP levels from serum samples collected from animals during in vivo study I, represented by the chemiluminescent signal, to determine whether differences in hSEAP could be measured in vivo N = 4. **(B)** hSEAP levels in serum samples from animals collected during in vivo study II, represented by chemiluminescent signal, to test the survival of transplanted gene-edited hESCs. N decreases as the animals are euthanized, according to the study design, but was always above 4. Columns represent the mean value, and error bars represent the standard deviation.

To test whether the hSEAP signal was strong enough for *in vivo* monitoring and obtain a timeline for the rejection of wildtype hESCs, an *in vivo* pilot study I was conducted (Figure 3). The successful detection of hSEAP would provide a timeframe for the rejection of wildtype hESCs in BALB/c mice. hSEAP analyses of serum from days 0, 1, 7, 10, and 15 showed a hSEAP signal for days 1 and 7 for both groups injected with low and high numbers of hSEAP-expressing hESCs (Figure 8A). Both samples from day 0 and wildtype hESCs showed no detectable signal. On days 1 and 7, the signal was significantly higher for the group injected with high numbers of hESC + hSEAP compared to that of the wildtype hESCs, according to Kruskal–Wallis analysis. The high number of hESC + hSEAP, furthermore, showed higher signals than the group injected with low numbers of cells. This confirms that differences in the numbers of hESCs are detectable *in vivo* and hereby highlights the capability of hSEAP as an *in vivo* biomarker. On day 10, the signal had decreased greatly, and it was depleted by day 14, indicating that the rejection of non-edited hESCs was initiated before day 10 in immune-competent mice.

The second *in vivo* study (*In vivo* study II) assessing the survival of gene-edited hESCs (Figure 4) showed no significant difference in hSEAP between the hESC + hSEAP, hESC + hSEAp+2xKO + mCD47, and hESC + hSEAp+2xKO + mCD47KO at the different time points, as determined by Kruskal–Wallis analysis (Figure 8B). On days 8 and 11, hSEAP levels decreased but were still above the detection limit. On day 15, the signal was no longer detectable, but the animals were kept in case a few hESCs survived and proliferated. On day 22, there was still no sign of growth, and the remaining animals were euthanized on day 25.

**FIGURE 9:**
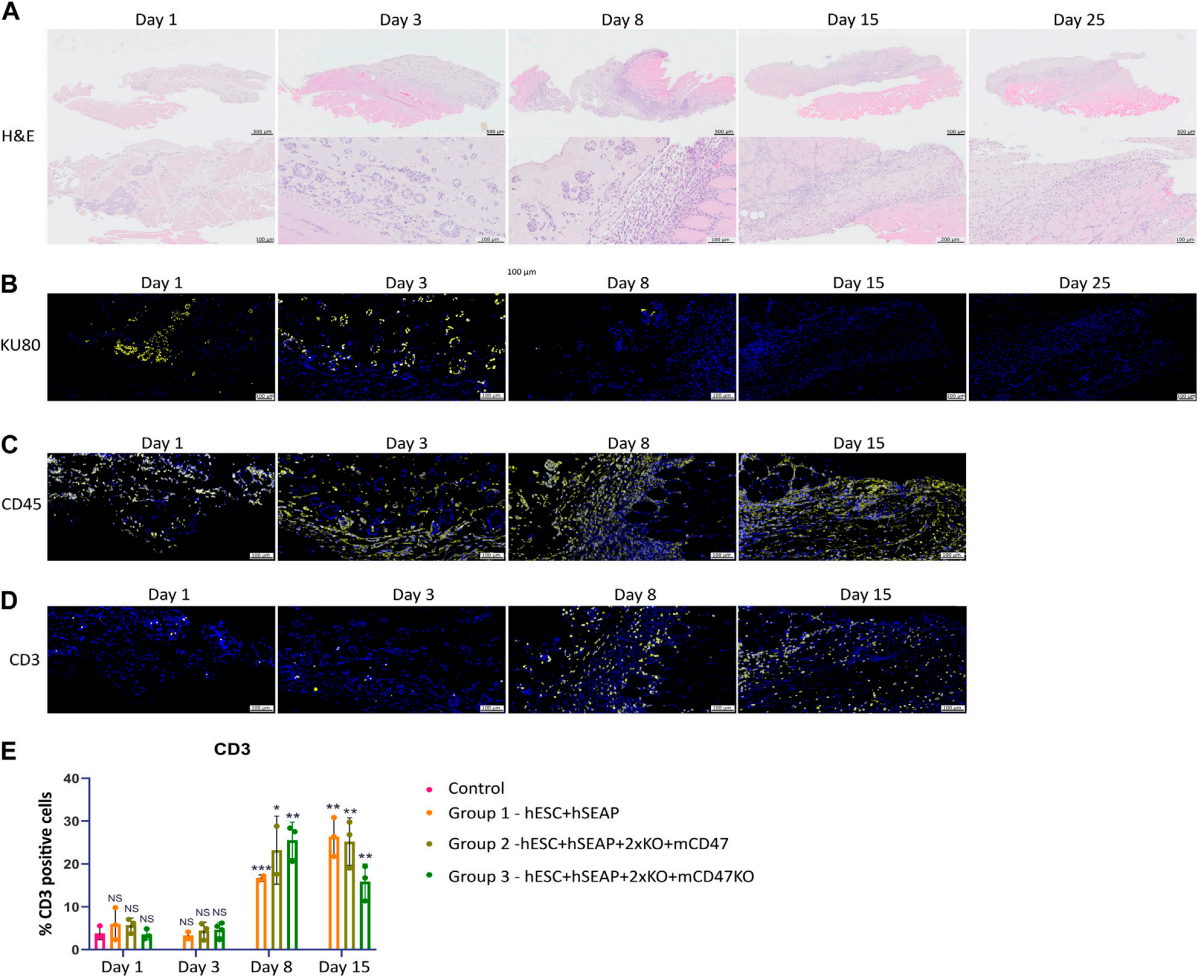
Histology of injection site N = 3. **(A)** H&E staining of the representative injection site from each of the three groups on different days. **(B–D)** Immunohistochemical staining of the representative injection site from each of the three groups on different days. **(B)** KU80 (yellow) and DAPI (blue), **(C)** CD45 (yellow) and DAPI (blue), and **(D)** CD3 (yellow) and DAPI (blue). **(E)** Quantification of % CD3 positive cells in the injection site compared to the % of DAPI, calculated from *3–5* images per injection site.

To examine the survival and immune response of hESCs, we performed immunohistochemistry to characterize the implantation site and regional lymph nodes at various time points. Figure 9 shows representative immunohistology for the transplant site of mice injected with hESC + hSEAp+2xKO + mCD47. The injection sites were readily visible with H&E staining. Cluster formation of cells with larger nuclei is present inside the injection site on days 1 and 3 (Figure 9A). On days 8 and 15, the clustered nuclei are no longer detectable in the injection site and randomly distributed smaller nuclei are present instead, indicating immune infiltration. Staining for the human nuclear protein KU80 confirms that the cluster formations seen in H&E are human cells (Figure 9B). No KU80-positive cells could be detected on days 15 or 25, and only a few positive cells were present in the injection sites on day 8. No human cells could be detected in the lymph node at any of the time points. To evaluate the contribution of leukocytes, the injection sites were stained with the general leukocyte marker CD45. Staining for the immune cells was only carried out up until day 15 since no cell clusters of hESCs were found after that point. All time points showed CD45-positive cells; however, the injection site showed obvious signs of immune infiltration on days 8 and 15 (Figure 9C).

CD3 staining to detect T cells indicated targeted rejection (Figure 9D). Furthermore, few CD3-positive cells should be present if the hESCs are cleared by apoptosis. However, on days 1 and 3, T-cell counts were similar to those of the control samples (Figure 9E). On days 8 and 15, T-cell counts significantly increased compared to control samples and samples from days 1 and 3, as determined by Kruskal–Wallis analysis (Figures 9D, E), suggesting rejection by an adaptive immune response.

## 4 Discussion

The current study presents the successful knockout of CIITA by CRISPR-Cas9 and knockout of B2M by the insertion of mCD47. Insertion of hSEAP in the safe harbor CLYBL could successfully be used as an *in vivo* marker to detect the survival of transplanted hESCs. Additionally, both hSEAP and mCD47 displayed stable expression in the gene-edited hESC during long-term culture and after differentiation.

The results of the *in vivo* studies did not support the hypothesis that knockout of MHC-I and II, along with mCD47 overexpression, can offer sufficient immune protection to prevent rejection in a xenogeneic host. Instead, it seemed that none of the transplanted hESC lines had survived past the 10th day of transplantation.

It would be necessary to sample more time points between days 3 and 10 to determine whether the hESC edits that were introduced reduced the rejection rate, potentially using the inserted firefly luciferase in addition to getting information on the graft growth through bioluminescence. Furthermore, to quantify a potential difference for the comparison of hSEAP data to histology, stereological sampling is necessary to calculate the number of hESCs and immune cells in the graft.

According to the available data, hESCs do not appear to be targeted or rejected during the first 3 days but appear to be multiplying, which implies that the hESCs are eliminated by the adaptive immune system. A study has previously reported cell loss by apoptosis 7 days after transplantation in PBS in syngeneic immunocompetent mice (Preda et al., 2021), which could explain the lack of difference in rejection between the three groups. However, several studies have effectively used Matrigel to subcutaneously transplant stem cells and their derivatives without graft loss for at least 2 weeks in immunocompromised mice (Oh et al., 2015; Gong et al., 2017; Yuan et al., 2017; Nagy and Nagy, 2018; Fu et al., 2019). This knowledge, in combination with the observed infiltration of T cells after day 8, led to the conclusion that the complete cell loss observed was not caused by apoptosis. Nevertheless, following hESC transplantation, some graft loss is unavoidable. This could provide a potential explanation for why every cell group was eliminated, as macrophages will clear dead cells, exposing parts of the dead cell on its surface to T-cells (Ferguson et al., 2011). Given the large number of proteins that distinguish humans from mice, it is possible that some of these presented proteins are perceived by T-cells as foreign, leading to the activation of an immune response even in the absence of MHC-I and MHC-II. Furthermore, it has been demonstrated that hSEAP stimulates the production of antibodies in mice but does not attract T cells (Mælandsmo et al., 2005), which may imply the necessity for a more diverse immune-protective strategy.

The use of immunocompetent animal models likely presents heightened rejection risks for hESCs compared to allogeneic and humanized models. Nonetheless, an immune-evasive hESC line within a xenogeneic environment, capable of conferring protection against immune rejection despite the presence of distinct proteins, has the potential to be protected in an allogeneic context as well. Furthermore, an immunocompetent model for hESCs offers a comprehensive insight into the immune potential, which, despite optimization efforts, remains incompletely elucidated in humanized animals. Moreover, instances have been documented where hESCs were not rejected in such models (Pizzato et al., 2024).

Most studies using gene-edited human stem cells *in vivo* have been conducted in immunocompromised mice (Dever et al., 2016; Roper et al., 2017; Métais et al., 2019; Park et al., 2019; Selvaraj et al., 2019; Dekkers et al., 2020; Bannier-Hélaouët et al., 2021). Therefore, only limited information on the potential of stem cell treatment and how these cells function in an immunocompetent host exists. The generation of immune evasive cells has mainly been tested *in vitro* for the activation of different immune cells (Torikai et al., 2013; Chen et al., 2015; Hong et al., 2017; Mattapally et al., 2018; Deuse et al., 2021), which only present information on a small aspect of the immune system. Other studies have transplanted stem cells to either allogenic animal models (Deuse et al., 2019), which, depending on the model, is restricted by its translational value to human stem cell transplants or humanized mice (Börger et al., 2016; Deuse et al., 2019; Xu et al., 2019; Norbnop et al., 2020), which can only partially mimic the human immune system. To our knowledge, only three papers have been published on transplanting edited human stem cells into immunocompetent mice, one of which assessed the survival of hESCs with B2M knockout for just 48 h in BALB/c mice (Lu et al., 2015). The second study presented tumor formation of B2M knockout hESCs in C57BL/6 mice lacking NK cells but did not present the number of animals treated and, thereby, no success rate (Wang et al., 2015). The third showed the survival of B2M knockdown hESCs in immunocompetent BALB/c mice (Deuse et al., 2011); the same researchers later expanded their strategy to include CIITA knockout and CD47 overexpression (Deuse et al., 2019). Based on these results, it was expected that both edited hESC lines presented in this study would show increased survival *in vivo*. The result that survival of xenogeneic transplantation in fully immunocompetent animals was not possible despite previous publications and the fact that the same editing strategy has shown long-term survival in immunocompetent BALB/c mice upon allogeneic transplantation (Deuse et al., 2019) highlights the problem with reproducibility.

Since there was no detectable difference in rejection between the hESC + hSEAP+2xKO + mCD47 and hESC + hSEAP+2xKO + mCD47KO lines, it is possible that the protection offered by the inserted mCD47 variant is less than with other splice variants of mCD47. Subsequent analysis of tissue from BALB/c mice showed the expression of splice variant 1 (Supplementary Figure S15), which differs by 21 amino acids missing in the linker region. This difference may, in part, help explain the lack of protection provided by mCD47. Deuse et al. demonstrated *in vitro* that for human cell lines to exhibit protective qualities against NK cells, the level of human CD47 must be raised by 3.5 times (Deuse et al., 2021).

In the present study, a more than 5-fold increase compared to endogenous mCD47 expression was detected by flow analysis, and it showed a mCD47 signal comparable to that of murine Min6 cells, which are known to express mCD47 (Zhang et al., 2020).

Since the reported 3.5-fold increase in hCD47 by Deuse et al. is compared to human induced pluripotent stem cells, which already possess endogenous expression of hCD47, the 5-fold increase of mCD47 reported in this study might not be enough for providing protection, considering that the fold change is compared to hESCs lacking endogenous mCD47 expression. This is in line with findings that hESCs with MHC class I and II knockout but no CD47 expression are rejected in humanized mice within 9 days (Hu et al., 2023). To investigate the protective effect of mCD47 and the targeting by NK cells in the double knockout cells, it would have been advantageous to conduct an *in vitro* experiment by co-culturing with NK cells. Such a study can additionally help to elucidate if a higher mCD47 expression is required for protection in a xenogeneic environment. In this case, a higher level of expression can be obtained by inserting additional copies in other loci.

To create an immune-evasive cell line, an alternative approach would be to overexpress additional immune-protective proteins alongside CD47. This would increase the cell’s barrier of defense and shield it from various immune cell types. Since NK cells are known to target cells lacking MHC-I specifically, one possibility would be to insert genes that are protective against them (Martinet and Smyth, 2015).

One tactic would be to overexpress HLA-E or HLA-G, which have less variation and would, therefore, require the production of fewer cell lines to cover the population’s immune profile. In humans, 98% of the population expresses one of the two HLA-E variants, E*01:01 and E*01:03 (Felício et al., 2014), and only two splice variants of HLA-G have been found to be expressed on the cell surface (Krijgsman et al., 2020). Hereby, the generation of four cell lines with homozygous expression could potentially match the variants present in the population.

Furthermore, HLA-E and G have been shown to enable the detection of infection by the immune system (Diehl et al., 1996; Kraemer et al., 2015). This may help avoid some of the issues that a fully immune evasive cell may present, such as serving as a harbor for the growth of tumors or the transmission of infections. For use in immunocompetent animals, the overexpressed MHC molecules would have to be species-specific.

The result that increasing the arm length can improve editing has previously been shown by increasing the arm length from 50 to 200 bp, which increased the efficiency by 9-fold (Li et al., 2014). Other studies have used 500–1,000 bp arms for the insertion of larger transgenes (Mahen et al., 2014; Merkle et al., 2015). For this reason, the editing design was changed during this study. The study showed that an advantage of longer arms is that they can work as a donor for more guides, potentially saving time for the design process. Instead of using longer arms, efficiency could be increased in other ways. Another approach could be to use a single homology arm, which has previously been shown to be successful for the insertion of a large fragment including both GFP and an antibiotic resistance gene using a 700 bp arm (Basiri et al., 2017). Both the knockout and insertions were made without detectable karyotypic changes and off-target effects. However, some differences in differentiation and PluriTest were observed in the edited and wildtype cell lines, which were, however, still characterized as pluripotent. Although these findings do not impact the primary objectives of this study, which involve assessing the survival of edited cells, they do suggest that the editing procedure could be further investigated. Specifically, exploring the potential impact of using a cell line with lower passage numbers and fewer rounds of editing on both PluriTest results and differentiation capability would be beneficial.

In conclusion, the insertion of hSEAP provided a stable expression, enabling the detection of cell viability *in vivo.* In the presented xenogeneic model, the transplanted gene-edited hESCs did not demonstrate immune evasion, but this challenge could potentially be addressed through the optimization of the editing strategy. The findings highlight obstacles that must be overcome for the generation of immune-evasive hESCs for xenogeneic *in vivo* testing. This cell line can work as a modeling system, providing information that is not accessible by studies using immune-compromised and humanized animal studies, as these fail to test the generated cell lines in a fully immunocompetent model.

## Data Availability

The original contributions presented in the study are included in the article/Supplementary Material; further inquiries can be directed to the corresponding author.
