# Supplementary material for: CRISPR-Cas9 immune-evasive hESCs are rejected following transplantation into immunocompetent mice
Source: Front Genome Ed. 2024 May 28;6:1403395. doi: 10.3389/fgeed.2024.1403395 (PMC11165197; doi:10.3389/fgeed.2024.1403395)
Supplement: Supplementary file 1 [file Table1.DOCX]

Supplementary Material


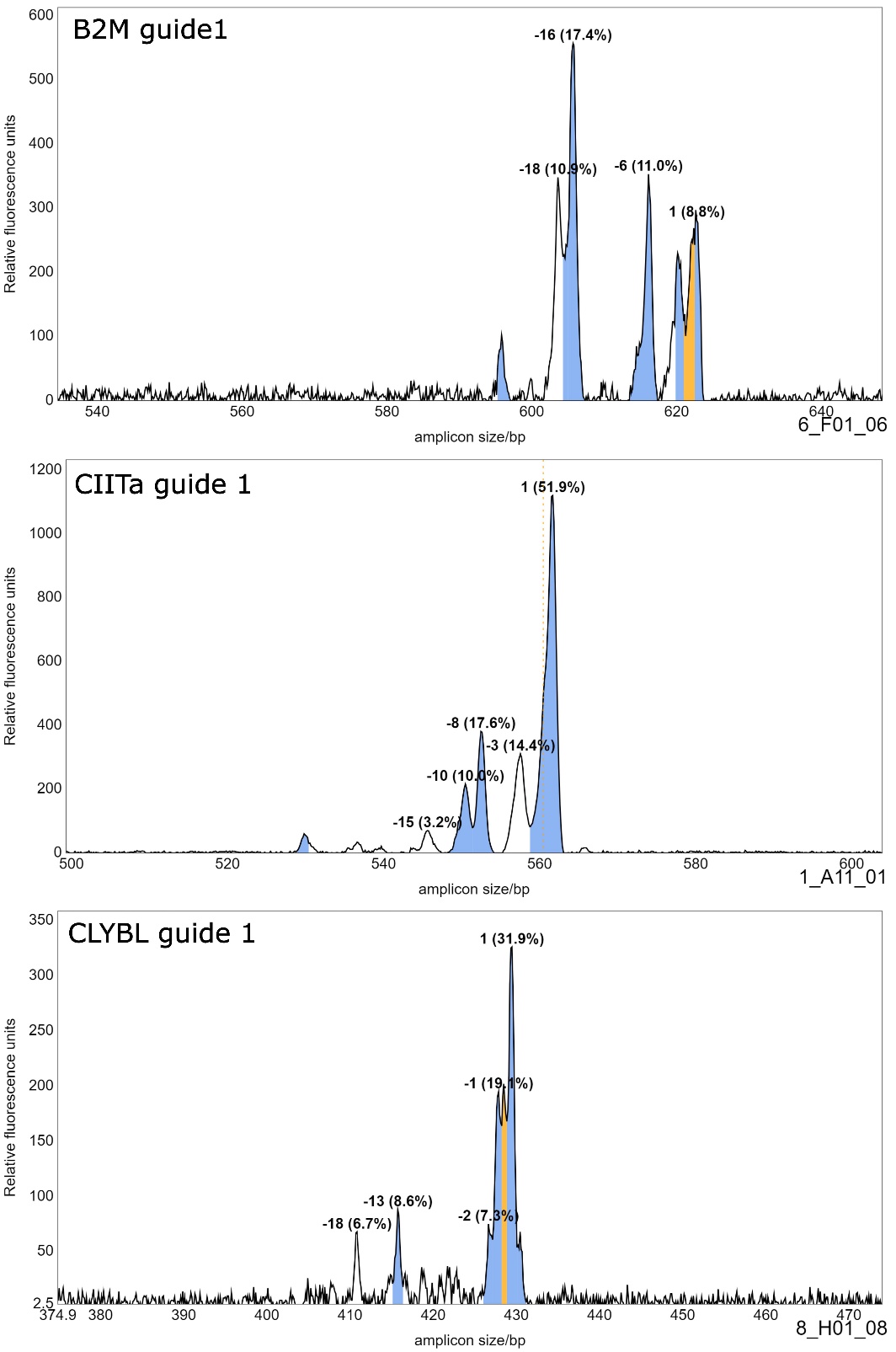


**Supplementary Figure 1**: IDAA profiles for cells nucleofected with CRISPR-Cas9 targeting B2M, CIITA or CLYBL. Indel events were detected by comparing ampliqon sizes of the nucleofected samples to wildtype DNA controls. Wildtype amplicon sizes, depicted in orange, were as follows: B2M - 622bp, CLYBL - 428bp, and CIITA - 560bp. The blue peaks represent the percentage of indels detected as a result of Cas9 cutting. The total percentage of indel formation is found by multiplying the blue peaks. These data are additionally provided as supplementary material for a pending publication.


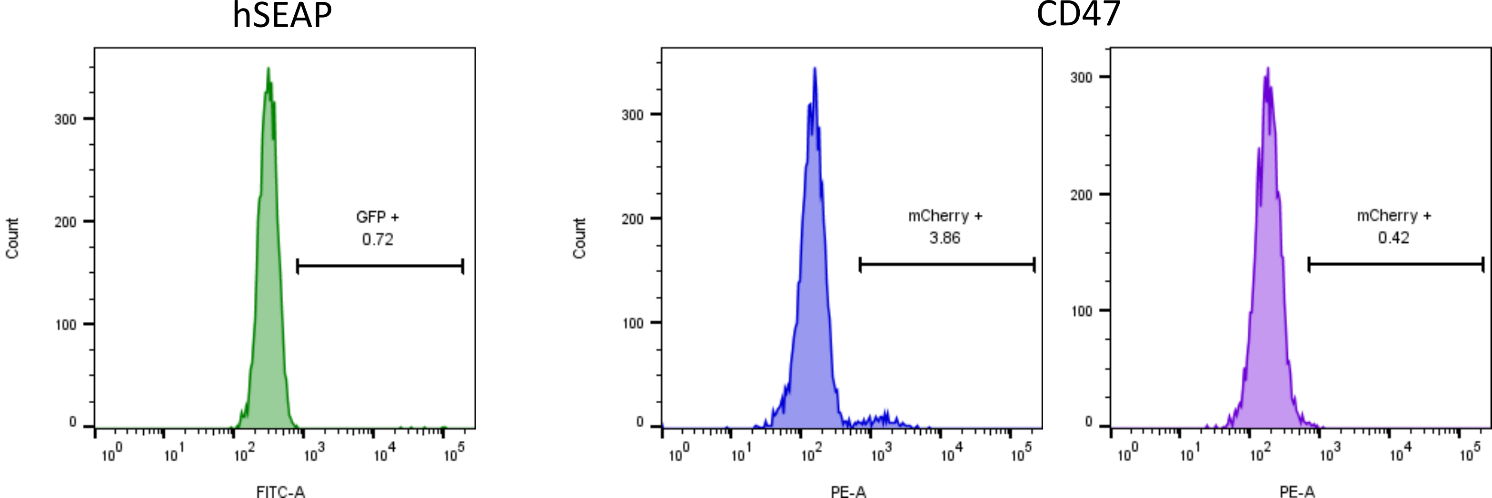


**Supplementary Figure 2**:

Flow cytometry analysis on bulk populations of edited cells prior to single-cell sorting. The edited cells were engineered to express either GFP (hSEAP) or mCherry (CD47) fluorescence markers. Gating was performed using a wildtype hESC negative control harboring non-integrated plasmid DNA. The gating identifies the percentage of cells expressing fluorescence due to successful plasmid integration. The CD47 edit was attempted with two different variants of CD47 inserted in identical plasmids. Subsequently, edited cells meeting the gating criteria were subjected to single-cell sorting for further downstream analysis.


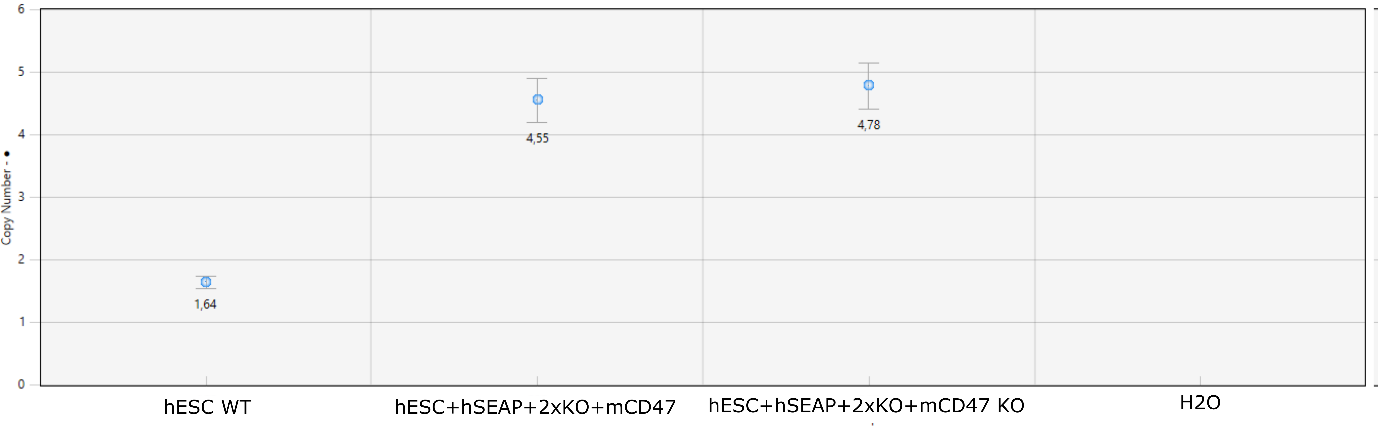


**Supplementary Figure 3**: Digital droplet PCR for generated cell lines. Digital droplet PCR targeting the UCOE site was made. Two copies of UCOE are naturally present in the hESC. The copy numbers are a total for both hSEAP and mCD47 insertion.


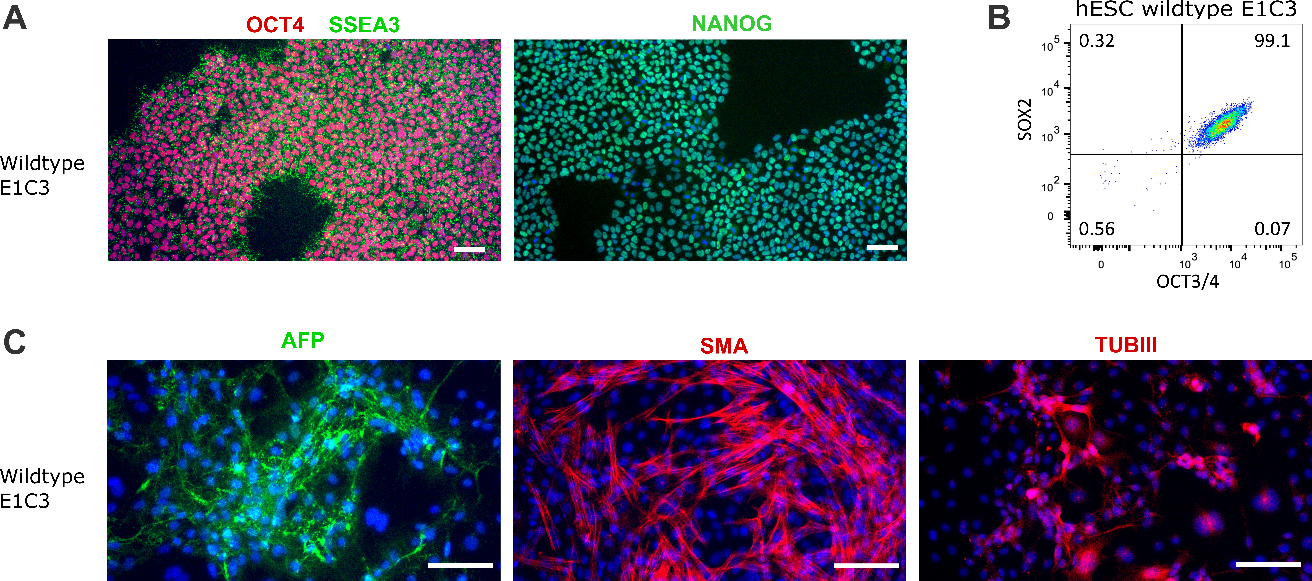


**Supplementary Figure 4**: Pluripotency of wildtype hESC E1C3. A. Immunocytochemical analysis was performed to detect pluripotency markers OCT4, SSEA3, and NANOG on wildtype E1C3 hESCs. Scale bars=100µm. B. Flow cytometry results depict the percentage of hESCs positive for OCT4 and SOX2 among the wildtype hESCs. Percentage shown in each quadrant. C. Immunocytochemical staining of gene-edited hESCs was conducted after 3 weeks of spontaneous differentiation. Markers representing each of the three germ layers were utilized to evaluate differentiation potential: smooth muscle actin (SMA) for mesoderm, beta Tubulin 3 (TUBIII) for ectoderm, and alpha-fetoprotein (AFP) for endoderm. Scale bars=100 µm.


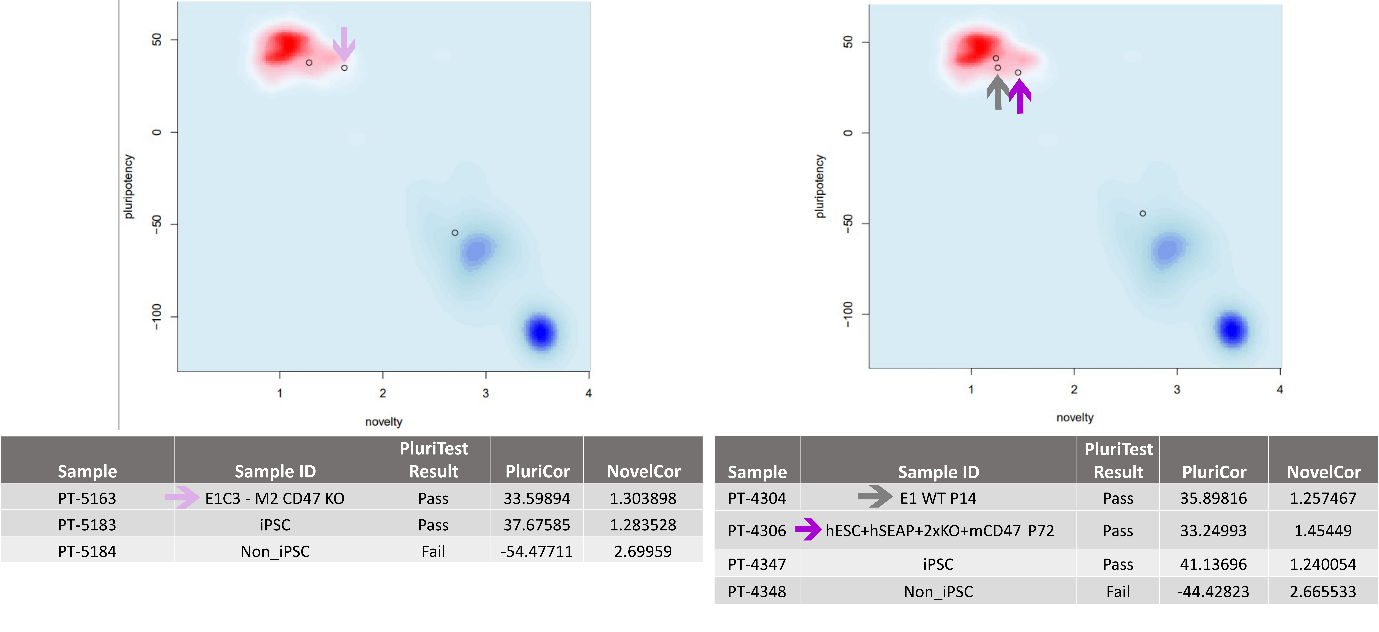


**Supplementary Figure 5**: PluriTest results for generated cell lines. PluriTest was performed by Thermo Fischer Scientific and analyzes the pluripotency of the generated cell lines by comparison to an established reference. Pluripotency is scored by two factors, novelty and pluripotency compared to either pass or fail the test. Pluripotency score indicates the expression profile of pluripotency markers. Novelty score indicates how similar the profile is to other pluripotent cells. In the plot, the red area shows the area in which the majority of pluripotent cells are located, the blue is for non-pluripotent cells. The tables present the values plotted and whether the cell lines have passed or failed the pluripotency test. Arrows point to the generated cell lines in the table and to their location on the plot.


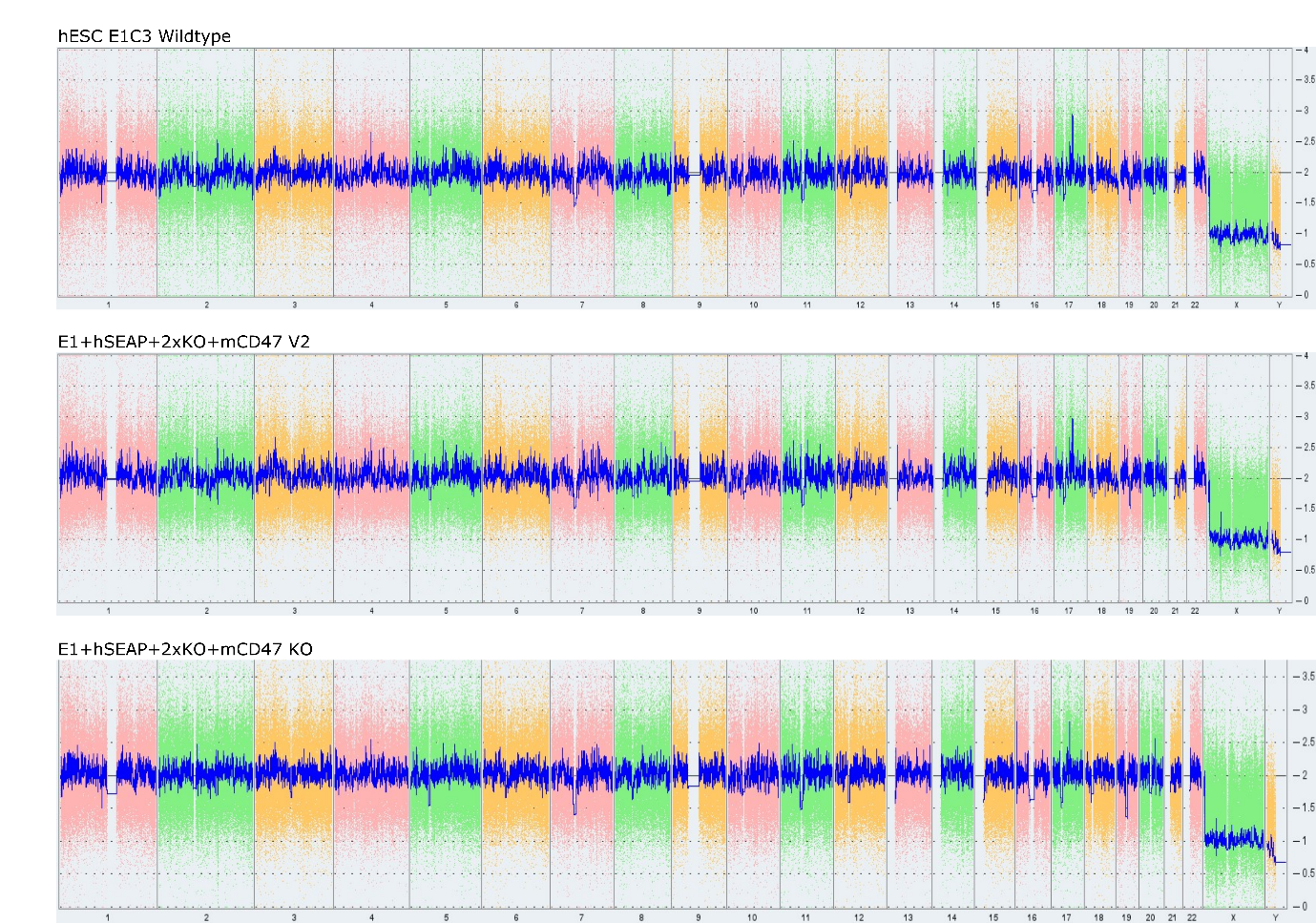


**Supplementary Figure 6**: Karyostat results. KaryoStat analysis showing a normal male chromosome for all cell lines. The blue line shows the copy number of each chromosome.


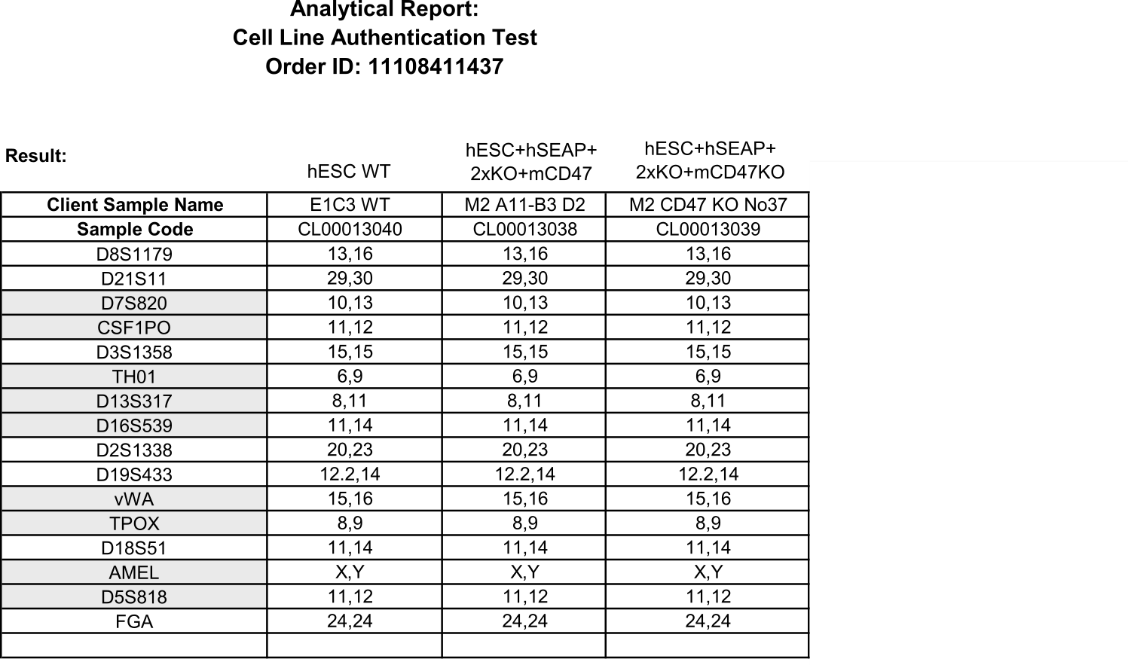


**Supplementary Figure 7**: Short tandem repeat (STR) analysis. Table presenting STR analysis results for DNA samples from each of the presented cell lines. STR performed by Eurofins Genomics.


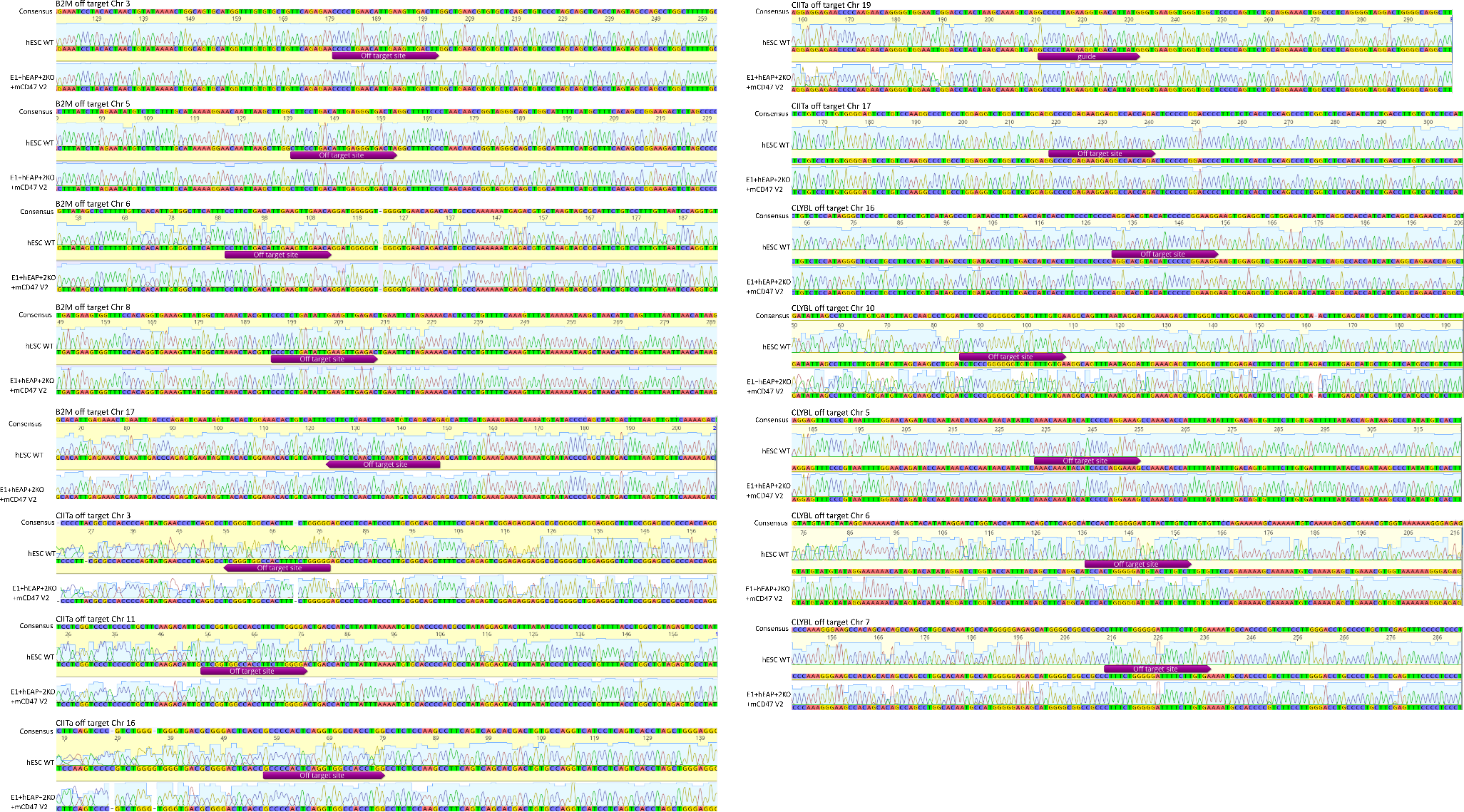


**Supplementary Figure 8**: Sequence of top 5 off targets predicted by CRISPRoff. Sanger sequencing for the top 5 off target sites for each of the three CRISPR-Cas9 designs targeting CLYBL, CIITA and B2M. Purple boxes indicate the part of the sequence in risk of off-targeted cutting. Wildtype hESC DNA is included for detection of potential alterations.


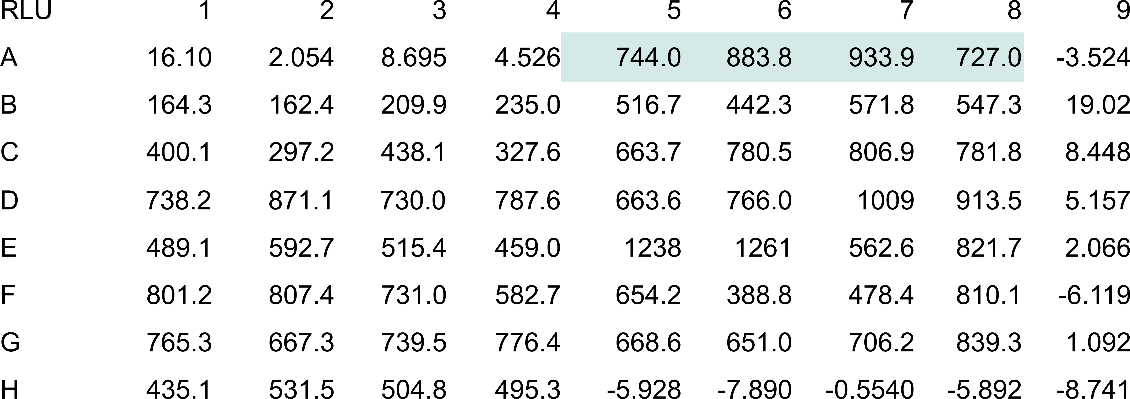


**Supplementary Figure 9**: Luciferase expression. Table showing luminescence signal of single clones upon treatment with D-luciferin. Cells were plated in quadruplicates. The hESC line E1+hSEAP+2xKO+mCD47 used in this study is marked in green. Column 9 contains non-treated cells as negative control.


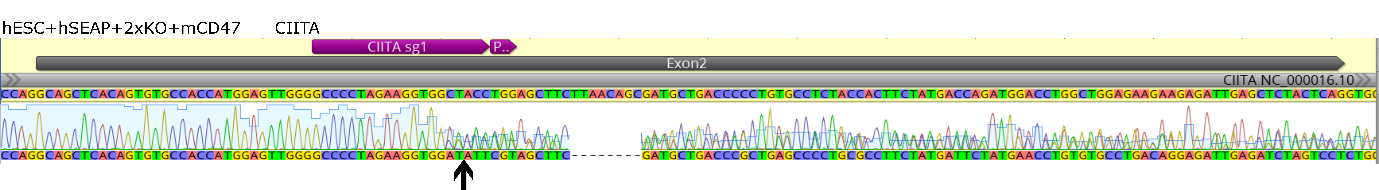
**Supplementary Figure 10**. CIITA sequence. Sanger sequence of the CIITA target site in E1+hSEAP+2xKO+mCD47. Black arrow indicates the cutting site by Cas9. The gRNA and PAM sequence is shown in purple.


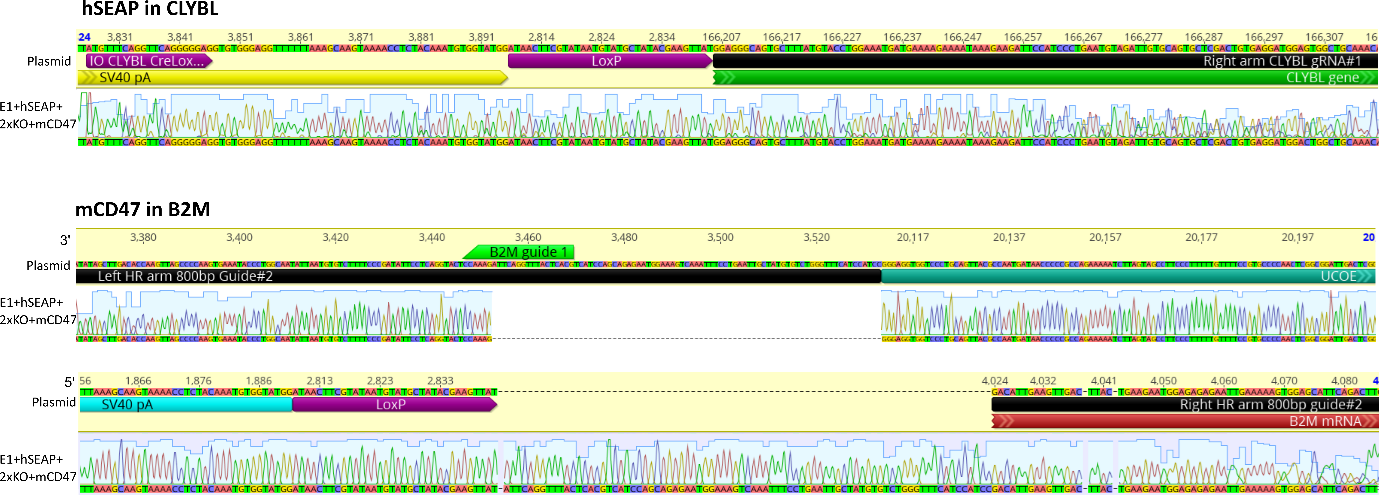


**Supplementary Figure 11**: Sanger sequence of 3’ end and 5’ end of inserted plasmids. The sequences from the two cell lines expressing murine CD47 were mapped to the plasmid sequence to detect potential changes. From the top: Sequence covering the 3’ end in the CLYBL site showing successful removal of the fluorescent cassette by mCre treatment for both cell lines. Sequence covering both the 5’ and 3’ end of the inserted murine CD47 plasmid show changes next to the homology arms corresponding to the cut site for the used CRISPR-Cas9 guide RNA, which is shown in green (B2M guide 1). In the 3’ end a deletion of 81 bp is observed, which for the 5’ results in insertion of missing 81 bp.


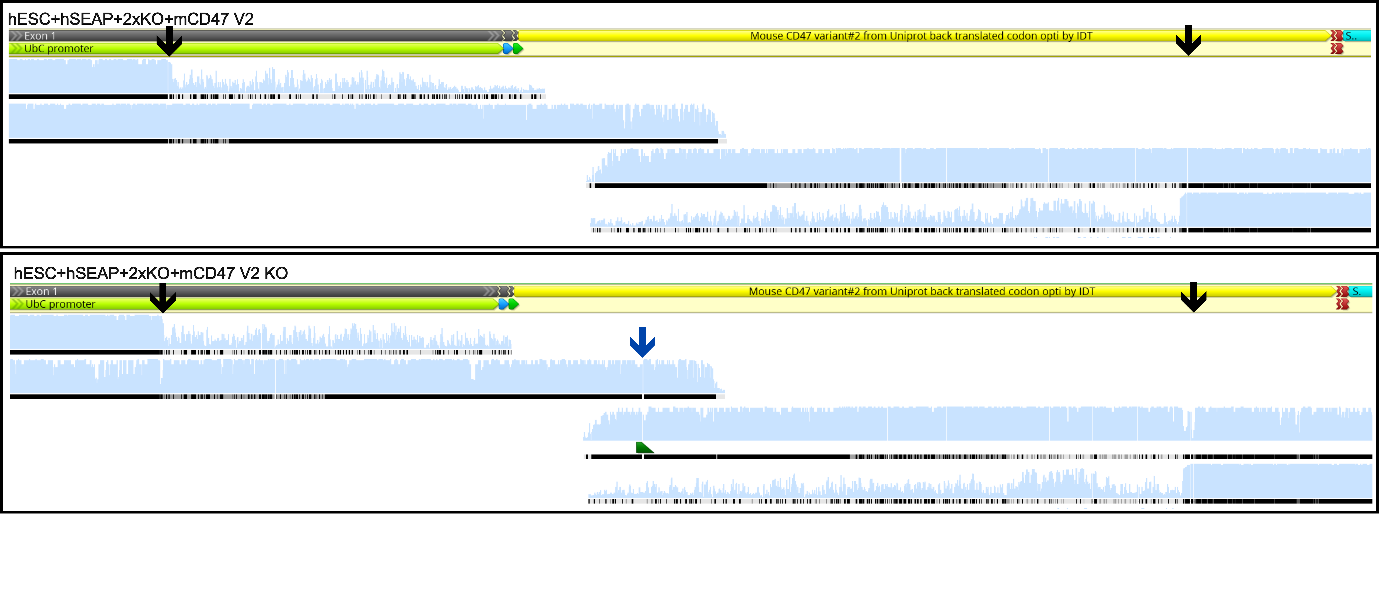


**Supplementary Figure 12:** Full CD47 sequence. The full sequence was obtained by primerwalk, performed by Eurofins genomics. The figure shows the plasmid design of the UbC promoter followed by murine CD47. Blue shading presents the sequencing signal, and the black lines show matching to the plasmid sequence. Black arrows indicate the start and end of the heterozygous deletion, evident by disruption of the black line. The blue arrow points to the cutting site for the CD47 KO.


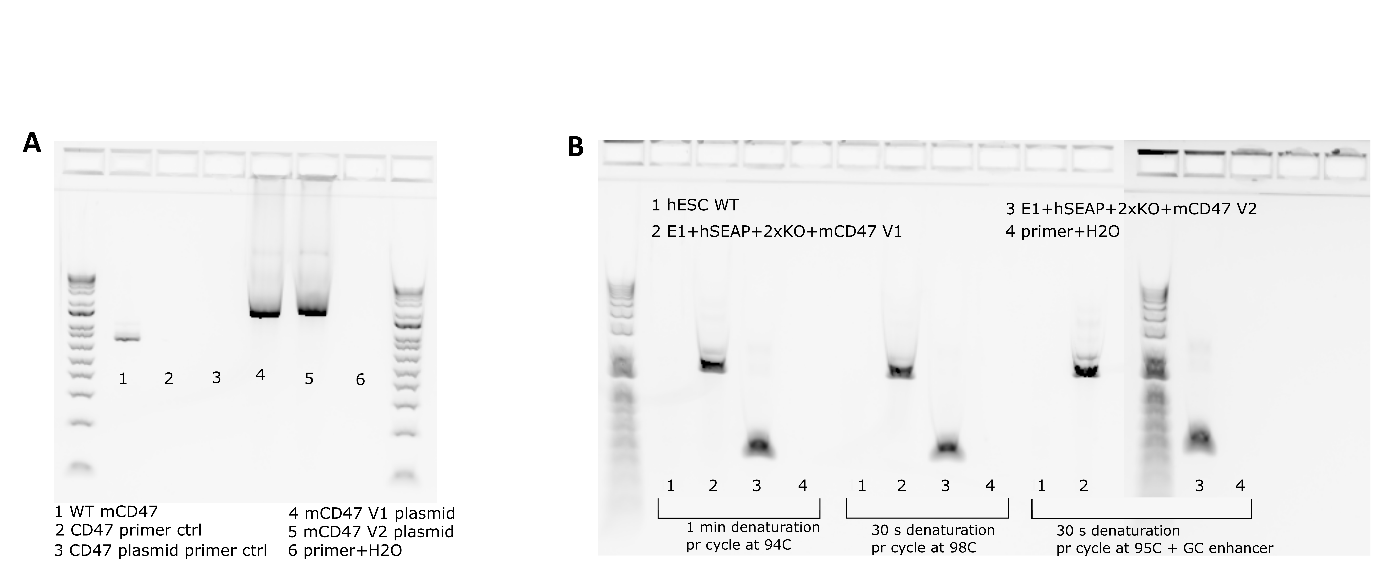


**Supplementary Figure 13**: PCR for murine CD47. A. PCR amplifying the UbC promoter and mCD47 in the plasmid. Primers used for long range PCR was used on wildtype DNA as control. B. PCR amplifying UbC and mCD47 was made for wildtype hESC and the two cell lines expressing each mCD47 splice variant. PCR was run at different settings, to investigate potential folding of the gDNA for mCD47 V2, but showed no difference in product size.


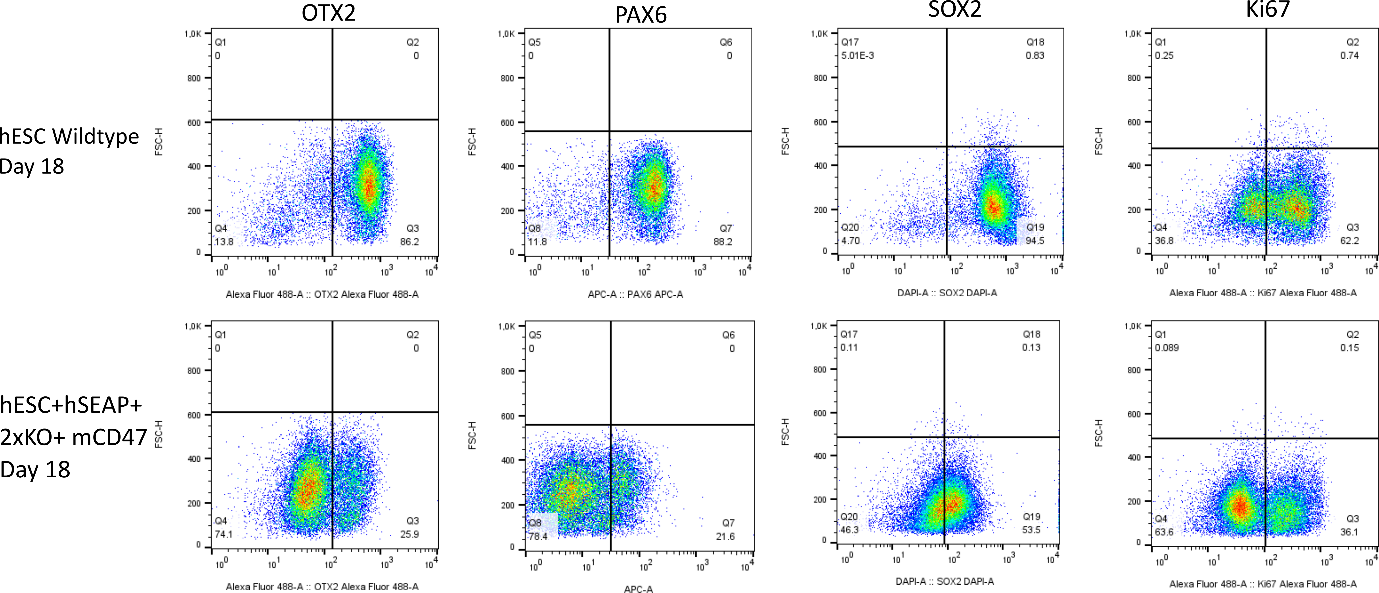


**Supplementary Figure 14**: Expression of neural markers after differentiation for 18 days. Flow cytometry analysis of wildtype control (upper panel) and gene edited cells (lower panel). Cells were stained with antibodies targeting OTX2, PAX6, SOX2, Ki67 and analyzed by gating to an undifferentiated hESC biological negative control. Percentage of positive cells are shown in each quadrant.


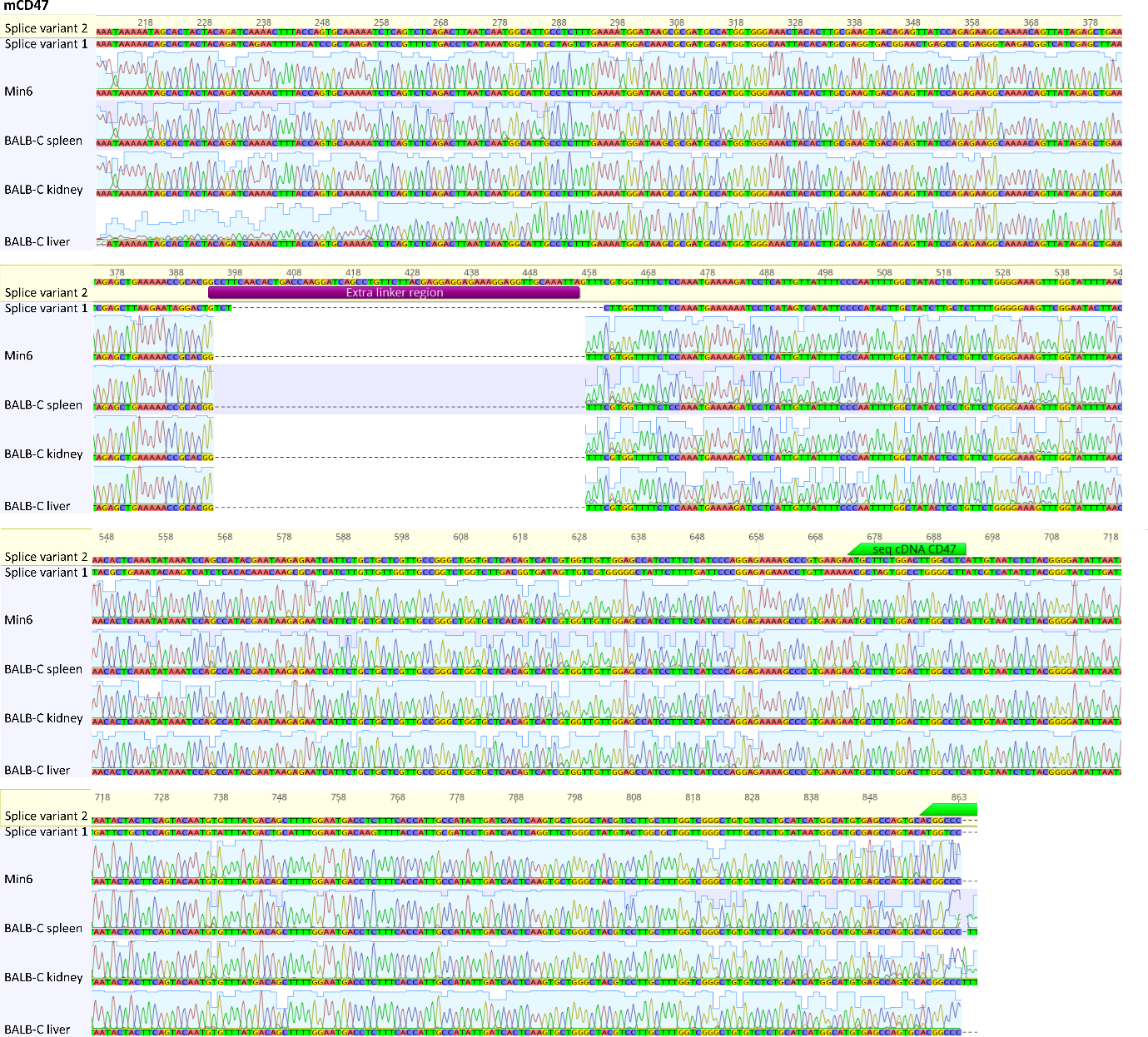


**Supplementary Figure 15**: Sequence of CD47 from different murine tissue. RNA was extracted from Min6 cells, liver, spleen and kidney from BALB-C mice. cDNA was obtained and the CD47 sequence was amplified by PCR and sequenced. Sequences were mapped to the plasmid sequences for mCD47 splice variant 1 and 2. Purple box indicate the extra linker region present in mCD47 splice variant 2, which is not present in any of the tissues.


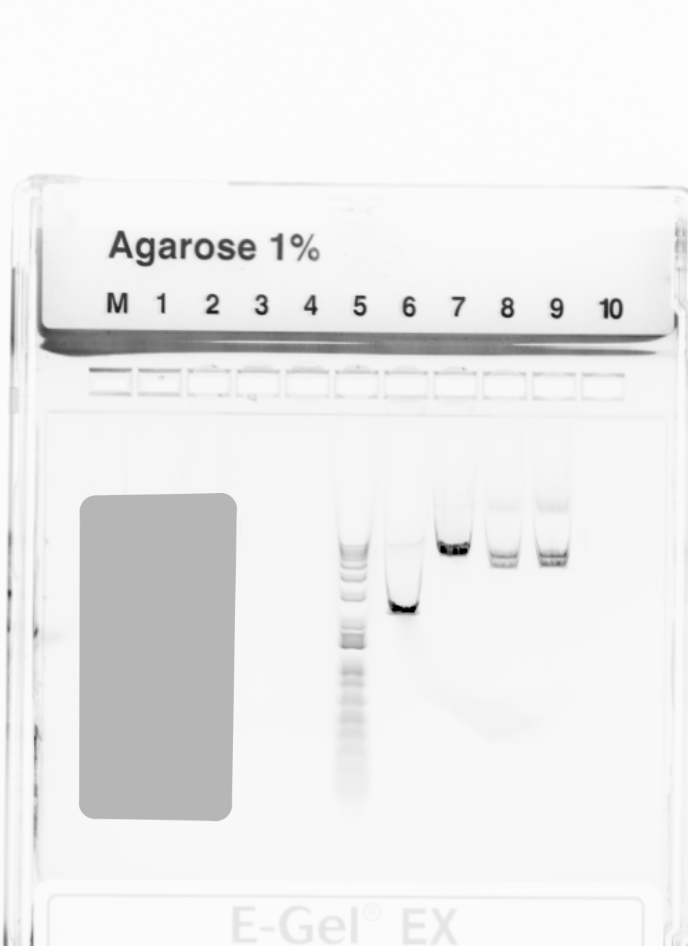

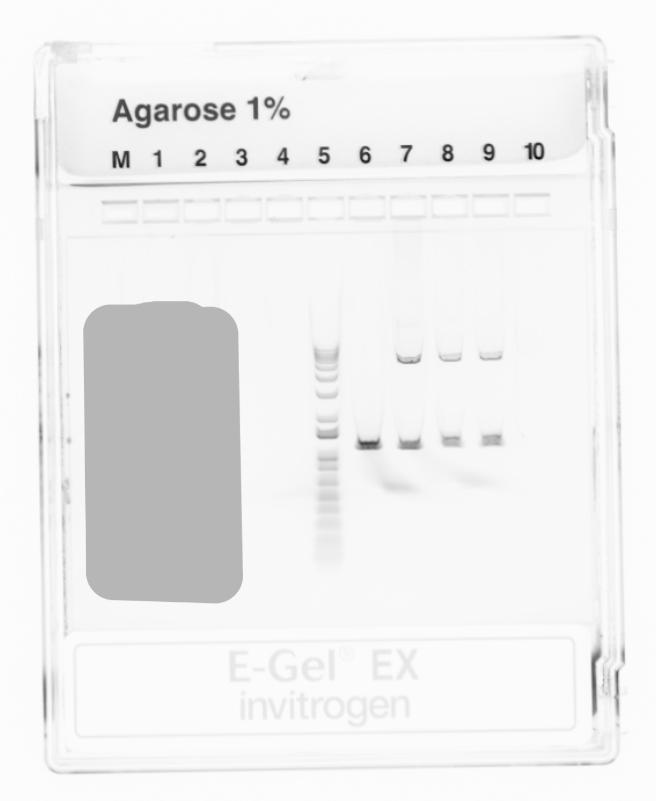


**Supplementary Figure 16**: Original agarose gel used for generating the cut out in Figure 6A. Right: CD47 insertion. Left: CLYBL insertion. DNA ladder: 1kb plus


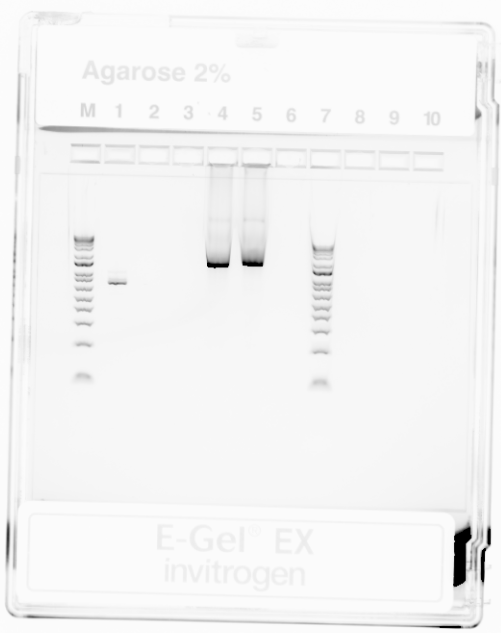

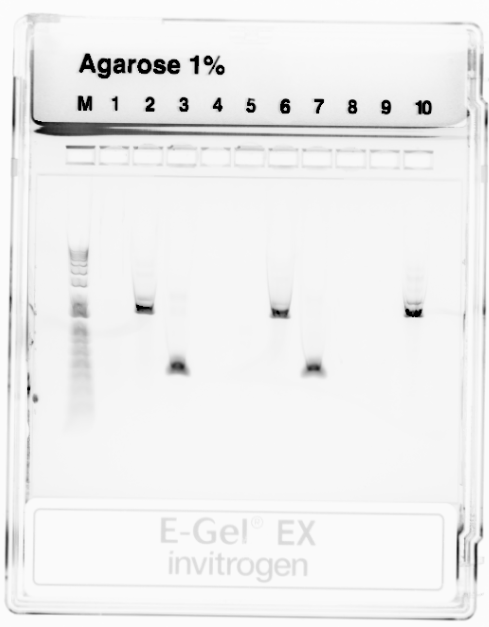

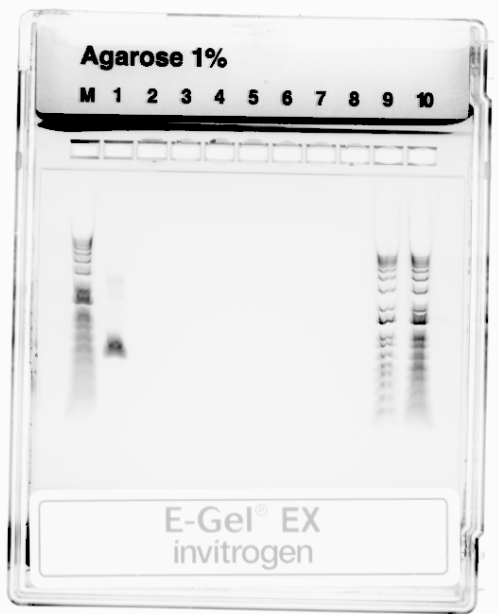


**Supplementary Figure 17**: Original agarose gel used for generating the cut out in Supplementary Figure 13 A and B. Right: A. Middle: Right B. Left: Left B. DNA ladder: 1kb plus
